# AlignScape, displaying sequence similarity using self-organizing maps

**DOI:** 10.3389/fbinf.2024.1321508

**Published:** 2024-01-26

**Authors:** Isaac Filella-Merce, Vincent Mallet, Eric Durand, Michael Nilges, Guillaume Bouvier, Riccardo Pellarin

**Affiliations:** ^1^ Life Sciences Department, Electronic and Atomic Protein Modeling Group (EAPM), Barcelona Supercomputing Center (BSC), Barcelona, Spain; ^2^ Institut Pasteur, Université Paris Cité, CNRS UMR 3528, Structural Bioinformatics Unit, Paris, France; ^3^ Laboratoire d'Ingénierie des Systèmes Macromoléculaires (LISM), Institut de Microbiologie de La Méditerranée (IM2B), Aix-Marseille Université, Centre National de La Recherche Scientifique (CNRS)-UMR 7255, Marseille, France; ^4^ Molecular Microbiology and Structural Biochemistry (MMSB), University of Lyon, Centre National de La Recherche Scientifique (CNRS)-UMR 5086, Lyon, France; ^5^ Laboratoire de Biologie et Modélisation de La Cellule, École Normale Supérieure de Lyon, CNRS, UMR 5239, Inserm U1293, Université Claude Bernard Lyon 1, Lyon, France

**Keywords:** self-organizing maps (SOM), sequence similarity landscape, protein sequence analysis, protein sequence visualization, human kinome, human GPCRs, type VI secretion system (T6SS)

## Abstract

The current richness of sequence data needs efficient methodologies to display and analyze the complexity of the information in a compact and readable manner. Traditionally, phylogenetic trees and sequence similarity networks have been used to display and analyze sequences of protein families. These methods aim to shed light on key computational biology problems such as sequence classification and functional inference. Here, we present a new methodology, AlignScape, based on self-organizing maps. AlignScape is applied to three large families of proteins: the kinases and GPCRs from human, and bacterial T6SS proteins. AlignScape provides a map of the similarity landscape and a tree representation of multiple sequence alignments These representations are useful to display, cluster, and classify sequences as well as identify functional trends. The efficient GPU implementation of AlignScape allows the analysis of large MSAs in a few minutes. Furthermore, we show how the AlignScape analysis of proteins belonging to the T6SS complex can be used to predict coevolving partners.

## 1 Introduction

The vast amount of biological data, in particular protein sequences, requires the development of methodologies for effectively displaying and analyzing the information they encompass. Sequence alignment serves as the starting point of any computational effort attempting to study the phylogeny of a given gene, as well as the computation of residue coevolution (de Juan et al., 2013), structure prediction (Jumper et al., 2021), functional inference (Emes, 2008; Yoon, 2009; Sanderson et al., 2023), and sequence clustering (Edgar, 2010; Fu et al., 2012). However, displaying and analyzing large sequence data sets is a challenging task, and it became desirable to develop frameworks for a convenient and compact manipulation of the data.

One possible way to display and analyze sequences is through phylogenetic trees, where each leaf represents an individual sequence, branches represent evolutive events, and nodes indicate the most recent common ancestor. Phylogenetic trees only use one dimension to arrange the input sequences. Thus, the arrangement of clades can be arbitrary, as each node can only produce a clade with two branches. Sequence similarity networks (SSNs) (Atkinson et al., 2009) have emerged as an alternative to address the limitations of trees. SSNs leverage the two-dimensional (2D) space’s topology, forming graphs where nodes are individual sequences and edges denote pairwise similarity relationships. This approach offers greater flexibility by connecting nodes with one, two, or multiple related nodes. Additionally, SSNs enable the analysis and display of significantly larger data sets compared to phylogenetic trees. Consequently, SSNs have the potential to uncover sequences that facilitate linking divergent clusters. This ability has proven particularly valuable for functional inference and sequence clustering (Copp et al., 2018). However, unlike phylogenetic trees, SSNs do not identify ancestral nodes or exhibit nested patterns associated with evolutionary descent. SSNs present two additional limitations: they yield different outcomes based on the choice of the distance cutoff, and they are mapped on an arbitrary space, relying on network visualization programs like Cytoscape (Shannon et al., 2003). Many additional methods have been developed to address specific tasks in sequence analysis. For instance, sequence clustering can be performed with hierarchical clustering algorithms (e.g., BLASTClust (NCBI News, 2023: Spring 2004|BLASTLab, n. d.) and mBKM (Wei et al., 2012)), greedy algorithms (e.g., cd-hit (Li et al., 2001)), or graph-based algorithms (e.g., ALFATClust (Chiu and Ong, 2022)). Functional inference, on the other hand, can be accomplished using methods based on sequence homology (e.g., BLAST (Altschul et al., 1990)), Hidden Markov Models (e.g., HMMER (Steinegger et al., 2019)), or deep neural networks (e.g., ProteInfer (Sanderson et al., 2023)).

Here, we introduce a Self-Organizing Maps (SOM) (Kohonen, 1982) approach to display and analyze an input multiple sequence alignment (MSA) in a compact and readable way. A SOM is an unsupervised machine learning technique intended to provide a low-dimensional representation of the input space while preserving the underlying topology on the output space. Hence, similar initial observations are mapped nearby. Previous studies have utilized SOM to cluster and classify protein sequences (Ferrán and Ferrara, 1991; Ferrán et al., 1994; Ahmad et al., 2008). However, these implementations were trained on small sets of sequences (less than 100) represented as amino acid frequency vectors. Moreover, the output maps lacked visual support, appearing as interconnected units with the input sequences mapped. Our approach, named AlignScape exploits the 2D output space to arrange the input sequences and the interpolated data. It provides a novel 3D graphical representation of large sets of aligned sequences, which are difficult to visualize using the standard phylogenetic trees and SSNs. Furthermore, AlignScape has the potential to address several crucial problems in sequence analysis, including sequence classification, functional inference, clustering, and gene coevolution. AlignScape demonstrates robustness to the choice of the input parameters and has a natural, non-arbitrary, and quantitative representation of the sequence similarity landscape. To assess AlignScape, we applied it to analyze the sequences from human Kinome and GPCRs as well as the proteins forming the bacterial Type VI Secretion System (T6SS).

## 2 Materials and methods

**FIGURE 1:**
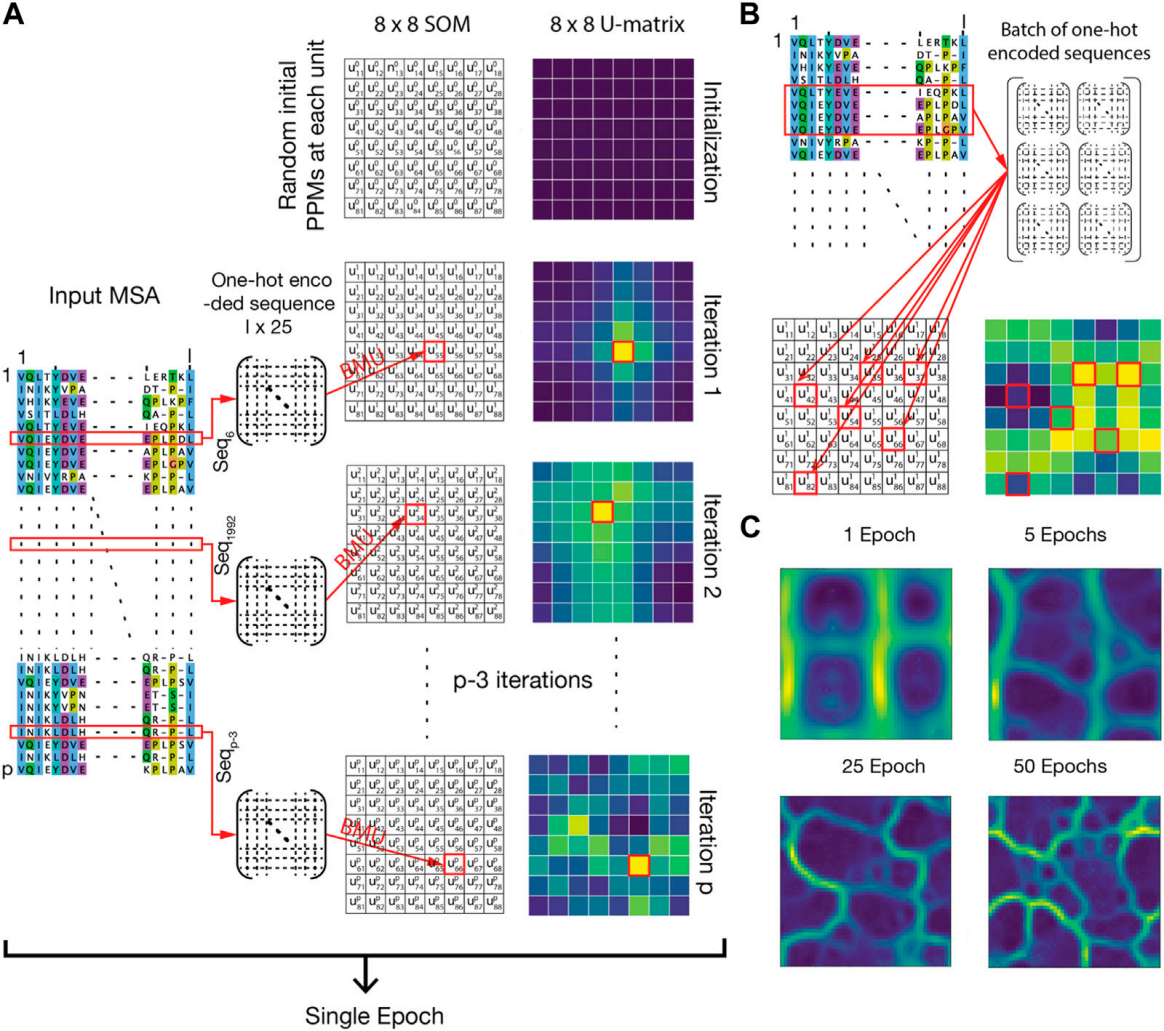
Description of the AlignScape methodology. **(A)** Each SOM unit is initially set to a random PPM. Iteratively, each sequence is randomly chosen from the MSA, is one-hot encoded, and assigned to its best matching unit (BMU). Then, the one-hot encoded sequence is accumulated to the PPMs of its BMU and their neighboring units. An epoch ends when all sequences are assigned. In the panel, the SOM and the corresponding U-matrix are represented as small 8 × 8 matrices for clarity. Units are represented by the symbol ukij where k stands for the iteration step, and i and j are the indexes of the unit. Red arrows and red squares highlight the BMU of the input sequences. **(B)** For efficiency, MSA processing occurs through batches of sequences randomly chosen from the MSA. **(C)** Evolution of the U-matrix at different epochs.

Here we describe the four fundamental steps used by AlignScape, and then we discuss a set of post-calculation analyses. AlignScape is novel, as it is developed to display sequence similarity landscapes, which are distinct from phylogenetic trees and similarity networks. It is built upon the self-organizing map (SOM) method and applied to MSAs. The core of the implementation and methodology is summarized as follows (Figure 1, for details, see Supplementary Methods).

• Step 1) Data representation: to compactly represent a set of aligned sequences, we employed a data structure known as the Position Probability Matrix (PPM) (Figure 1A). A PPM is a multidimensional array of frequencies that an MSA site is assigned to a residue type or a gap. The PPM of a single protein sequence is a one-hot encoding matrix.
• Step 2) Initialization: the output SOM is a periodic 2D grid with a predetermined number of units. Each SOM unit corresponds to a PPM. Initially, units consist of randomly generated PPM (Figure 1A).
• Step 3) Training: iteratively, each sequence from an input MSA is one-hot encoded and assigned to its closest unit, referred to as the best matching unit (BMU). Then, the input sequence is accumulated to the PPMs of its BMU and the neighbor units, optimizing the overall similarity of all involved units (Figure 1A). To maximize efficiency, the iteration can be structured in batches of input data instead of single sequences (Figure 1B). The epochs are complete iterations that cover all sequences of the input MSA. Finally, the training phase ends after a desired number of epochs.
• Step 4) Visualization and interpretation: after the training, the SOM is depicted as a unified distance matrix, known as U-matrix (Figures 1A–C). The U-matrix provides a convenient representation of the MSA’s similarity landscape by reporting the PPM distance (PPMd) between adjacent SOM units and therefore characterizing the MSA’s underlying topology. The PPMd is a new metric that we introduced to compute pairwise distances between PPMs, and it was derived to perform efficiently on GPUs (see Supplementary Methods). Generally, the U-matrix is organized into “basins” of similar sequences and “barriers” of distant sequences. Occasionally, funnels link neighboring basins through barriers, and a single basin may connect with multiple others. This landscape representation of the sequences is novel with respect to other methods.

**FIGURE 2:**
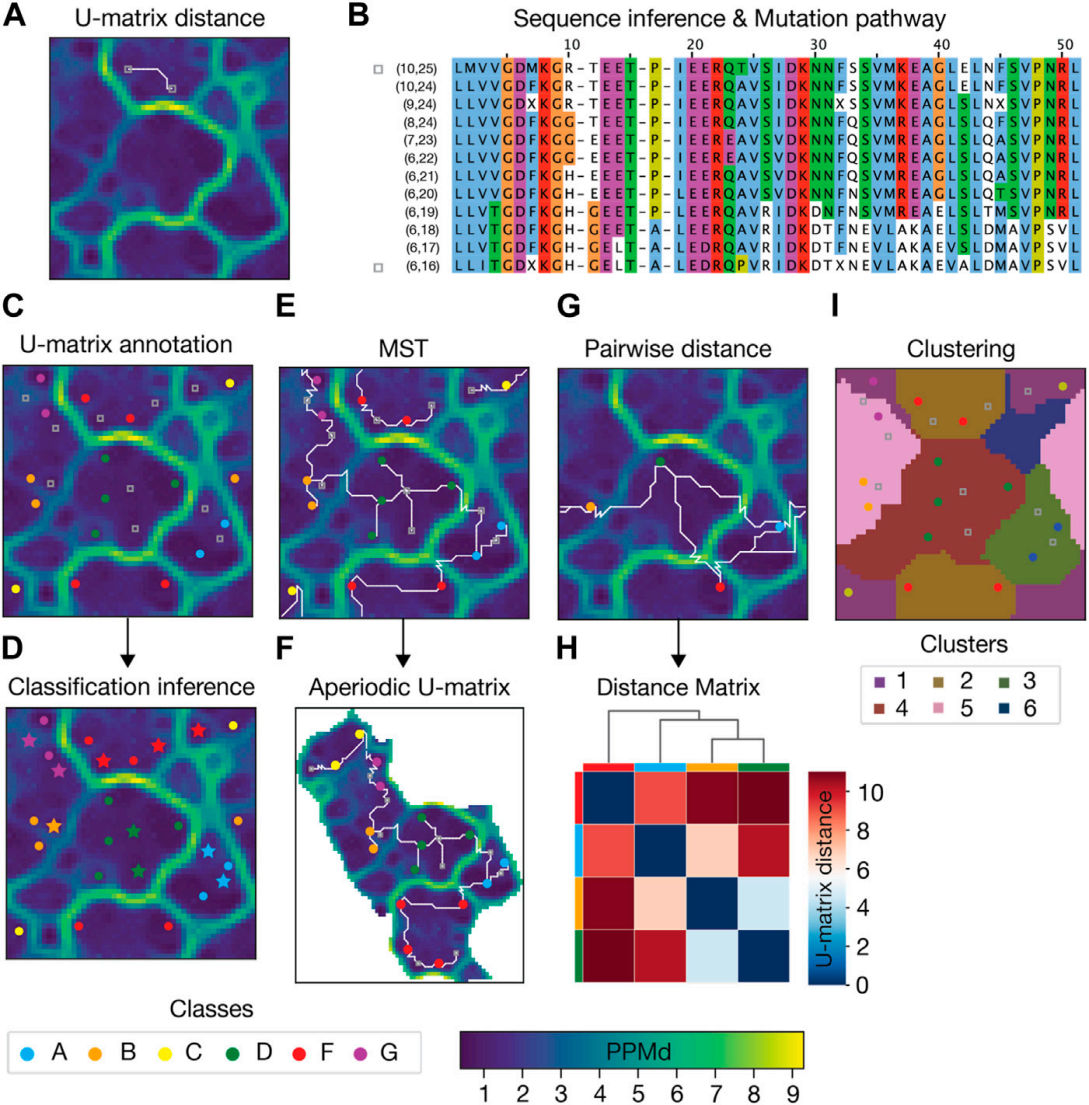
Example of AlignScape analysis. **(A)** U-matrix distance: the U-matrix distance between two input sequences is depicted as the shortest path connecting their BMUs (highlighted as gray squares). **(B)** Mutation pathway: the mutation pathway between two input sequences (sequences from Panel A) is the MSA of the sequences inferred from the units that the shortest path between the input sequences traverses. MSA sequences are labeled with their corresponding U-matrix unit indexes. The mutation pathway’s starting and ending sequences are highlighted with gray squares. **(C)** U-matrix annotation: the BMUs of sequences with prior classification data are color-coded according to their sequence class. BMUs of sequences without prior classification data are highlighted with gray squares. **(D)** Classification inference: annotated sequences are used to infer the class of non-annotated sequences. Sequences whose class was inferred are highlighted with colored stars. **(E)** Minimum Spanning Tree (MST): the MST connecting all mapped sequences represented as the set of shortest paths with minimal total length and no cycles. **(F)** Aperiodic U-matrix: the MST connectivity from Panel E is used to transform the periodic U-matrix from Panel E into an aperiodic U-matrix. **(G)** Pairwise U-matrix distances: U-matrix distances between all mapped sequences. **(H)** Distance matrix: hierarchically clustered distance matrix and corresponding dendrogram computed with the distances from panel **(G)**. Colored boxes between the matrix and the dendrogram indicate the mapped sequences. **(I)** U-matrix clustering: clustered U-matrix where each unit was color-coded according to the cluster to which it belongs.

Several post-calculation analyses can be carried out on a SOM and its corresponding U-matrix (Figure 2). Here below we list a number of analysis that we implemented and used in the examples presented below.

• Computing the U-matrix distance between sequences. The 3D representation yielded by the U-matrix allowed us to define a new distance between sequences which is the length of the shortest path connecting their BMUs (Figure 2A).
• Annotating the U-matrix for Genetic and Functional Inference. Each sequence from the input MSA is assigned to a unique BMU. This mapping can carry additional information about the sequence phylogenesis or function, which can be annotated in the U-matrix unit (Figure 2C). Sparse annotation of the U-matrix can be used to infer the genetic or functional classification of non-annotated sequences (Figure 2D).
• Representing the relationship between the input sequences as a Minimum Spanning Tree (MST). The MST connects all BMUs of input sequences without cycles, and with a minimal total U-matrix distance between them (Figure 2E).
• Transforming the U-matrix from periodic to aperiodic grid. The standard periodic U-matrix representation, where the units at the boundaries of the SOM are neighbors, can be confusing, as it breaks the continuity of basins and barriers. To address this issue, an aperiodic U-matrix can be computed based on the MST (Figure 2F). This aperiodic U-matrix ensures the topographical connection of the MST while guaranteeing spatial integrity.
• Clustering the U-matrix. U-matrix boundary regions, whose assignment to neighboring basins is ambiguous, can be assigned to a single basin using the agglomerative clustering algorithm (Figure 2I). This clusters all units such that all pairs of units belonging to the same cluster are maximally close, and those belonging to different clusters are maximally distant. Importantly, in our implementation, the user is not required to predefine the number of clusters.
• Inferring the sequence of a given U-matrix unit. Each SOM unit has an associated PPM that can be decoded into a sequence that was not input. This is achieved by returning the sequence with the highest joint probability of individual amino acids (Figure 2B). Therefore, the inferred sequences are interpolations performed by AlignScape using the initial MSA data.
• Inferring Mutation Pathways. To infer the mutations between two input sequences, we calculate the shortest path connecting their BMUs. The PPMs of the units along this path and their corresponding inferred sequences allow for retrieving an MSA that highlights the most likely series of mutations between the two input sequences (Figure 2B).
• Calculating the distance matrix between input sequences. The U-matrix distance enables the computation of pairwise distance matrices for any set of input sequences. These matrices can be hierarchically clustered, and their resulting dendrogram used to visualize the proximity between U-matrix units and groups of units (Figure 2H).
• Calculating the correlation between the distance matrices of two different genes. When two gene MSAs are annotated with sets of sequences with the same taxonomic or genetic information (i.e., the organism or the gene cluster they belong to), one can compute the Pearson correlation between the two corresponding distance matrices as a measure of coevolution (Supplementary Figure S1). This assumes that coevolving genes produce SOMs with similar topologies.

## 3 Results

### 3.1 Human kinome

Kinases belong to the phosphotransferase superfamily, a group of enzymes that catalyze protein phosphorylation. The human Kinome contains more than 500 kinase genes, classified as typical kinases and atypical kinases, based on their structure (484 proteins comprising a total of 497 typical kinase domains and 29 atypical kinases (Modi and Dunbrack, 2019)). One-third of these genes belong to the dark Kinome (Berginski et al., 2021), kinases whose functional role remains unclear.

**FIGURE 3:**
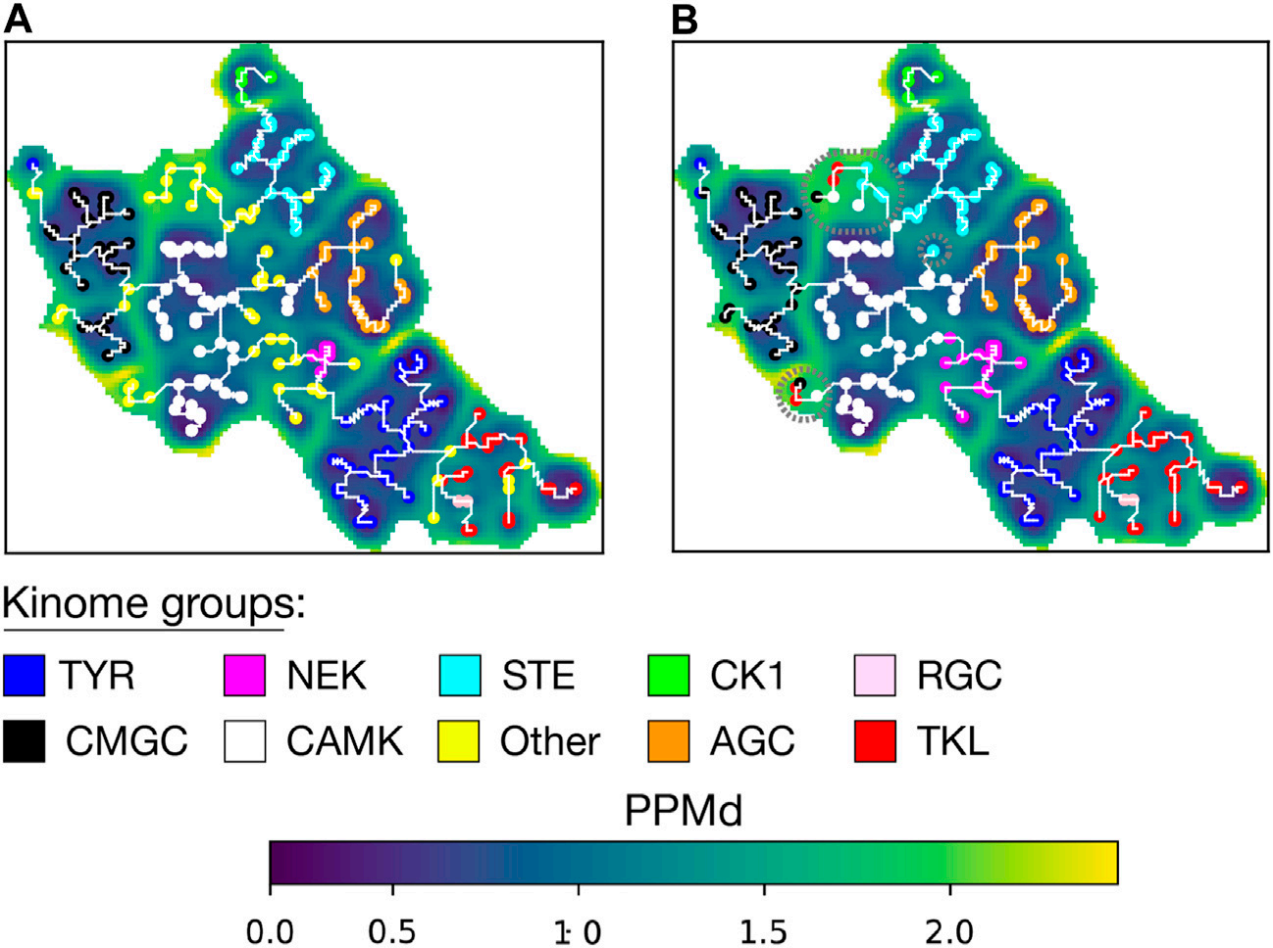
Aperiodic U-matrix of the kinome. **(A)** Sequence similarity landscape of the human Kinome represented with an aperiodic U-matrix. Each sequence from the input MSA was mapped to its BMU, with BMU colors indicating the sequence’s kinase group. The input MSA of 497 sequences had a length of 369 sites. The training of the SOM was refined over 200 epochs. The SOM size was set to 90 × 90. The SOM calculation took 8 min on a Tesla T4 GPU with 15 GB memory on Google Colab. The MST analysis took 9 min in a single CPU on Google Colab. **(B)** Aperiodic U-matrix from panel A featuring the BMU of the sequences from the unclassified group (‘OTHER') color-coded according to the inferred groups. The inference was performed using a k-nearest neighbors algorithm with k = 1. Sequences in high PPMd regions whose group inference was identified as ambiguous were highlighted with gray dashed circles.

To validate AlignScape, we computed and analyzed the sequence similarity landscape of the human Kinome. To do so, we utilized a high-quality structural-based MSA of the 497 typical kinase domains, from which a phylogenetic tree was computed in the past (Modi and Dunbrack, 2019). Notably, all aligned sequences were annotated by their typical kinase group (AGC, CAMK, CK1, CMGC, NEK, RGC, STE, TKL, TYR) and an additional group for unclassified kinases (OTHER) (Manning et al., 2002). The U-matrix arranges kinases belonging to the same group together (Figure 3A). It is important to note that a U-matrix unit can be associated with more than one sequence from the input MSA, and no unit in the Kinome U-matrix had sequences from different typical kinase groups. The MST recapitulates the Kinome phylogenetic tree (Modi and Dunbrack, 2019). For instance, the branching between TYR and TKL and the inclusion of RGC into the main branch of TKL is reproduced in both the MST and the phylogenetic tree. We also reproduce the early splitting of CMGC’s branch into two clades and the proximity between CAMK and NEK, separated in both representations by a small unclassified group. Importantly, the OTHER sequences are mostly found at the edge between groups or at terminal branches of the MST.

Next, we aimed to infer the group for the unclassified kinases. To do so, we first assessed the robustness of the inference analysis (Supplementary Figure S2A) by randomly removing 25% of the labels from the annotated units (149 units for training and 50 units for testing corresponding to about 330 and 110 sequences, respectively). We obtained a mean accuracy for the inference of 99% when it was based on one k-neighbor. We then inferred the kinase group of the OTHER sequences (Figure 3B and Supplementary Table S1). For the 66 sequences of the OTHER group, the assignment was coherent with the position in the MST, with the exception of some sequences in the high PPMd regions of the U-matrix.

As a test, we inferred a mutation pathway between two input sequences (Supplementary Figure S3A). The pathway consists of a set of sequence variants interpolated by the SOM (not included in the input MSA), which smoothly connect the two input sequences (Supplementary Figure S3D). The pathway can be interpreted as the minimum set of BLOSUM62 transformations applied to the first input sequence to reach the second. The analysis predicts that some mutations occur simultaneously along the pathway, while others are asynchronous, suggesting compensatory mutations occurring at different places on the pathway.

### 3.2 Human GPCRs

G protein-coupled receptors (GPCRs), the largest family of cell surface receptors (Lagerström and Schiöth, 2008), are crucial targets in numerous diseases (Zalewska et al., 2014; Alhosaini et al., 2021). They play a vital role in sensing a wide range of extracellular signals, including photons, small molecules, and large peptides. These signals are subsequently transduced into intracellular signaling cascades, which ultimately regulate key cellular functions. Despite their sequence diversity, GPCRs share a common structure of a transmembrane domain (TMD), consisting of seven transmembrane helices and three extracellular and intracellular loops (Yang et al., 2021).

To establish a comprehensive and accurate classification of human GPCRs, V. Cvicek et al. (Cvicek et al., 2016) conducted a comparative analysis between the GRAFS (Fredriksson et al., 2003) and IUPHAR (Sharman et al., 2013) classifications. To do so, they computed a structure-based MSA of the TMD for all human GPCR sequences. These MSA sequences were annotated based on the groups and subgroups defined in both GRAFS and IUPHAR classifications, including rhodopsins (A-α, A-β, A-γ, A-δ), secretin receptors (B), adhesion receptors, glutamate receptors (C), frizzled receptors, olfactory receptors, bitter taste receptors (Taste2), vomeronasal receptors, and an additional subgroup for unclassified rhodopsin GPCRs (A-other). To benchmark AlignScape, we used the V. Cvicek et al. MSA to compute and analyze the sequence similarity landscape of the human GPCRs.

**FIGURE 4:**
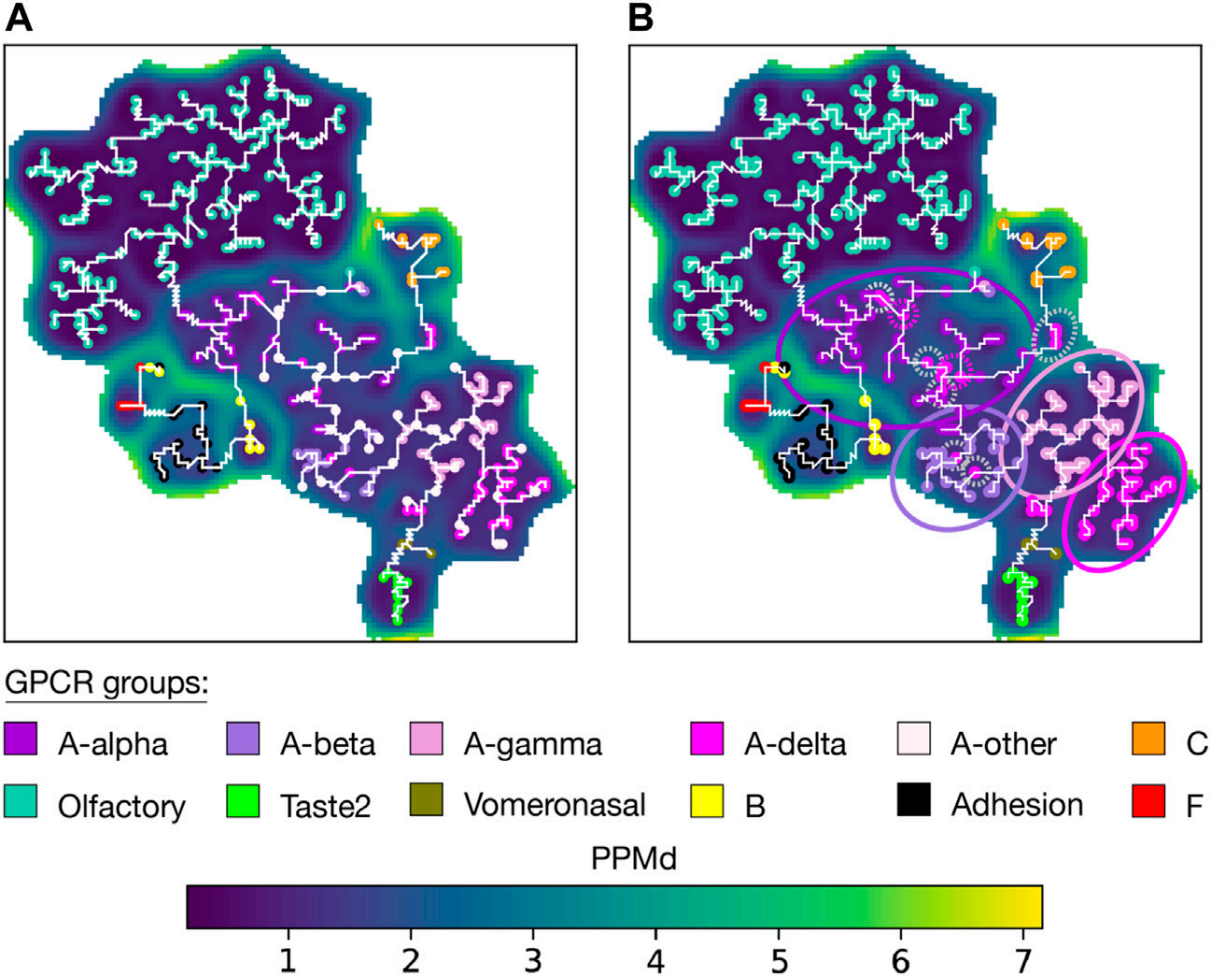
Aperiodic U-matrix of GPCRs. **(A)** Sequence similarity landscape of the human GPCRs represented with an aperiodic U-matrix. Input MSA sequences were mapped to their BMUs, which were colored according to the group of their sequences. The input MSA of 828 sequences had a length of 980 sites. The training of the SOM was refined over 200 epochs. The SOM size was set to 90 × 90. The SOM calculation took 33 min on a Tesla T4 GPU with 15 GB memory on Google Colab. The MST analysis took 40 min in a single CPU on Google Colab. **(B)** Aperiodic U-matrix from panel A featuring the BMUs of the sequences from the A-other group color-coded according to the inferred subgroups. The inference was performed using a k-nearest neighbors algorithm with k = 1. A-alpha, A-beta, A-gamma, and A-δ branches were highlighted with circles accordingly colored. Gray dashed circles highlight A-δ sequences within the A-α or A-β branches. Pink dashed circles were used to highlight sequences of the A-other that were assigned to the A-δ subgroup despite being located in the A-α or A-β branches.

The U-matrix arranges GPCRs belonging to the same group together (Figure 4A). The U-matrix topology reveals a central basin composed of all A GPCRs. Four MST branches stem from this basin: 1) A branch with all olfactory sequences grouped into a basin with low PPMd values, indicating a high degree of similarity among these sequences. 2) A branch with all C sequences. 3) A branch with all vomeronasal sequences that subsequently extends to Taste2 sequences. 4) A branch traversing four basins containing B sequences, Adhesion sequences, F sequences, and a mixture of the three groups. Notably, only 6 units out of the total units with an associated sequence (1.4%), had a mixture of sequences from different GPCR subgroups. These specific units have a combination of A-δ with either A-γ, A-β, or A-α sequences.

Unlike the phylogenetic tree constructed by V. Cvicek et al., our MST lacks a root, making direct comparison between the two representations challenging. Based on previous data suggesting that C is the oldest GPCR group (Krishnan et al., 2012), V. Cvicek et al. set the tree root within C. Concordantly, the U-matrix arranged C sequences within a basin encircled by high PPMd values (Figure 4 and Supplementary Figure S4). Q9UBS5 sequence, stood out as a highly dissimilar C sequence, making it a potential candidate for outgroup placement when rooting a phylogenetic tree. The MST also agreed with the phylogenetic tree and the IUPHAR classification by directly connecting Vomeronasal and Taste2 sequences with A sequences. Similarly, they agree in connecting the olfactory and A sequences. These observations contrast with the GRAFS classification, where Taste2 sequence were grouped within F, and where neither vomeronasal nor olfactory sequences were included due to the absence of a “clear phylogenetic relationship to any of the main families." In both the tree and the MST, the precision of the subgrouping of A sequences is acceptable. Only some A-δ sequences were placed outside the A-δ clade/branch. Namely, a few A-δ sequences are observed within the A-α branch, and the A-β branch (Supplementary Table S2).

Next, we wanted to infer the A subgroup of the sequences annotated as ‘A-other' (Figure 4B and Supplementary Table S3). First, we assessed the robustness of the inference (Supplementary Figure S2B) by randomly removing 25% of the labels from the annotated units and then inferring their group/subgroup with the remaining 75%. This validation yielded a mean accuracy of 97% when the inference was based on one k-neighbor. We then infer the subgroup of all sequences in the ‘A-other' subgroup. This enabled us to clearly identify four distinct branches within the A basin. However, we observed the inclusion of some inferred A-δ sequences within the A-α branch (Supplementary Table S2).

### 3.3 Type VI secretion system (T6SS)

T6SS is a macromolecular machine prevalent in Gram-negative bacteria. Pathogens deploy T6SS within human hosts due to its capacity to target prokaryotic symbiotic organisms (Jana and Salomon, 2019; Allsopp et al., 2020) and eukaryotic host cells (Sana et al., 2015; Crisan and Hammer, 2020). T6SS is a contractile injection system that operates as an inverted bacteriophage, functioning from within the attacking bacteria to the external environment (Figure 6A). It exhibits homologies with bacteriophages and other secretion systems (Durand et al., 2012; Taylor et al., 2018), suggesting a mosaic evolution in which T6SS co-opted pre-existing functional blocks from various cellular machines (Denise et al., 2020). Phylogenetic studies classified the system into four types known as T6SS^i^ to T6SS^iv^ (Boyer et al., 2009; Barret et al., 2013a). T6SS^i^ has been further classified into six subtypes: i1, i2, i3, i4a, i4b, and i5. A canonical T6SS is composed of 13 core components encoded in a gene cluster and structurally organized into three subcomplexes: the baseplate (BP), the membrane complex (MC), and the contractile tail complex (TC) (Figure 6A). The BP consists of TssE, TssF, TssG, and TssK proteins and is tipped by the spike, a pointed conic structure made of VgrG proteins. The MC includes TssJ, TssL, and TssM proteins. The TC comprises TssB, TssC, and Hcp proteins, along with two auxiliary proteins: TssA, assisting the assembly of the TC, and ClpV, responsible for recycling the TC components for subsequent rounds of secretion.

AlignScape was used to compute and analyze the similarity landscape of all 13 T6SS^i^ core components. Unlike the kinases and GPCRs examples above, the MSAs of T6SS^i^ genes lack structural validation, and prior classification into T6SS^i^ subtypes was very sparse. Consequently, this presented a more difficult scenario for benchmarking AlignScape.

**FIGURE 5:**
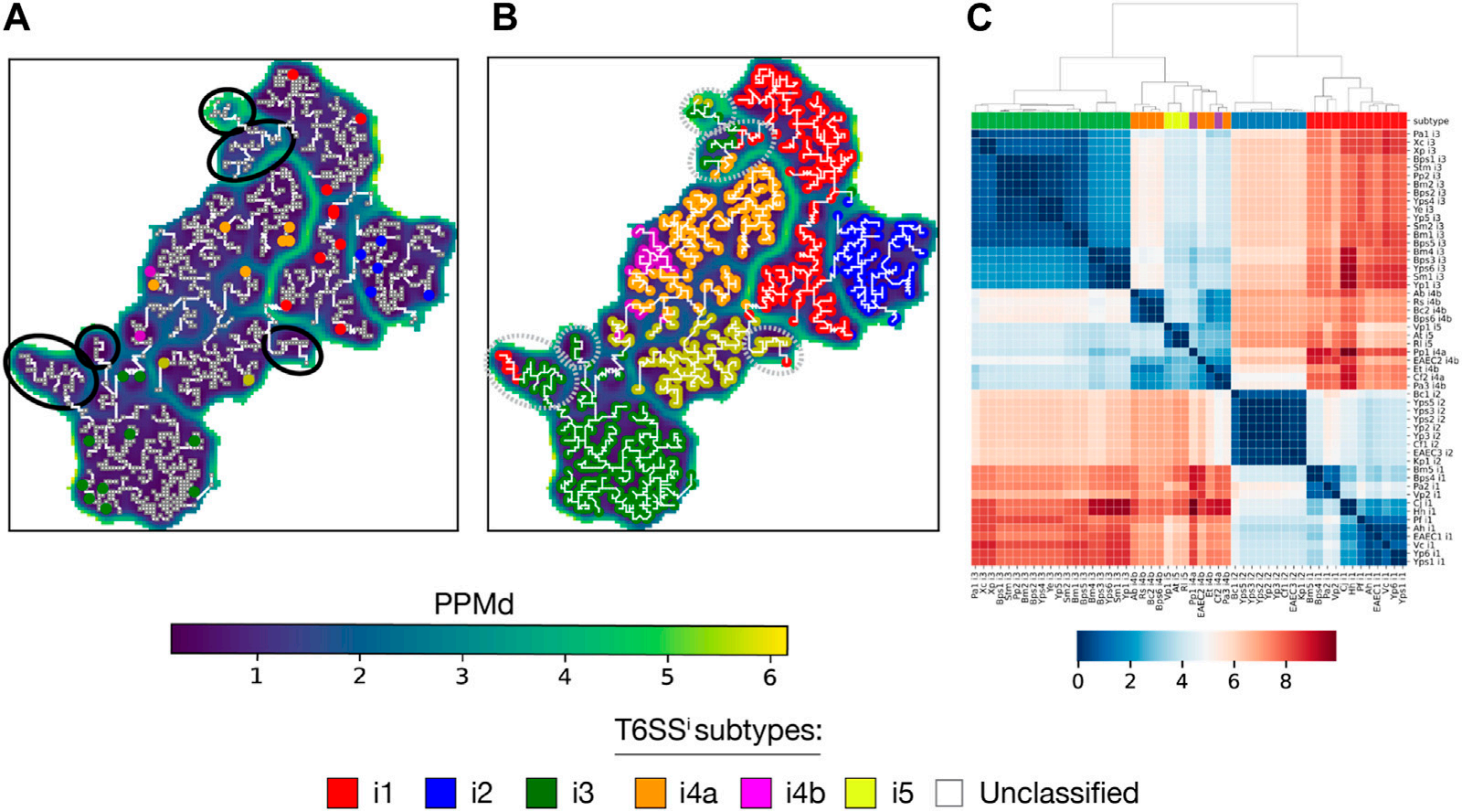
Aperiodic U-matrix and distance matrix of TssB family. **(A)** Sequence similarity landscape of the TssB gene represented with an aperiodic U-matrix. Input MSA sequences with prior information on their T6SS^i^ subtype were mapped to their BMUs, which were colored according to the sequence subtype. Gray squared units along the MST correspond to the BMUs of the sequences without prior classification data. Black circles denote basins without classified sequences. The input MSA consisted of 2,916 TssB homologs and had a length of 203 sites. The SOM size was set to 90 × 90, and the training of the SOM was refined over 200 epochs. The SOM calculation took 9 min on a Tesla T4 GPU with 15 GB memory on Google Colab. The MST analysis took 1 h and 23 min in a single CPU on Google Colab. **(B)** Aperiodic U-matrix from panel A, where colored BMUs substituted the gray squared BMUs according to the inferred T6SS^i^ subtype. The inference was performed using a k-nearest neighbors algorithm with k = 1. Gray dashed circles highlight basins without sequences with prior classification data, hence basins containing sequences for which inference was ambiguous. **(C)** TssB distance matrix computed with the sequences with prior classification data (sequence acronyms reported in Supplementary Table S4). Colored boxes between the matrix and the dendrogram indicate the T6SS^i^ subtype of each sequence.

We began with the SOM of TssB, one of the most conserved T6SS genes. Despite the few prior data on subtype classification, the TssB U-matrix arranged the sequences from the i1, i2, i3, and i5 subtypes into separate basins (Figure 5A and Supplementary Figure S5A). As for the i4a and i4b sequences, they were arranged within the same basin but distributed across different regions, with i4b sequences occupying a small area in the lower-left part of the basin. Importantly, none of the annotated units had sequences from different T6SS^i^ subtypes. Additionally, the U-matrix presented several basins entirely composed of unclassified sequences, from which taxonomical data provided limited insights into their origin. This observation aligns with the understanding that T6SS evolution is largely independent of species evolution (Boyer et al., 2009). Upon inferring the subtypes of unclassified sequences (Figure 5B, Supplementary Figure S5B and Supplementary Table S6), the assignment to basins to the i1, i2, i3, and i5 subtypes clearly distinct, as well as the distribution of the i4a and i4b sequences within their own basin. Conversely, the basins of unclassified sequences displayed a mixture of subtypes, indicating a need for further analysis to accurately infer their subtypes.

Next, we computed the TssB distance matrix (Figure 5C). The corresponding dendrogram reaffirmed the previously observed grouping of subtypes in different U-matrix basins. Notably, the dendrogram displayed patterns that concurred with evolutionary relationships among T6SS^i^ subtypes, such as the early bifurcation into two clades: one with i1 and i2 sequences and the other with i3, i4a, i4b, and i5 sequences (Boyer et al., 2009; Barret et al., 2013a).

**FIGURE 6:**
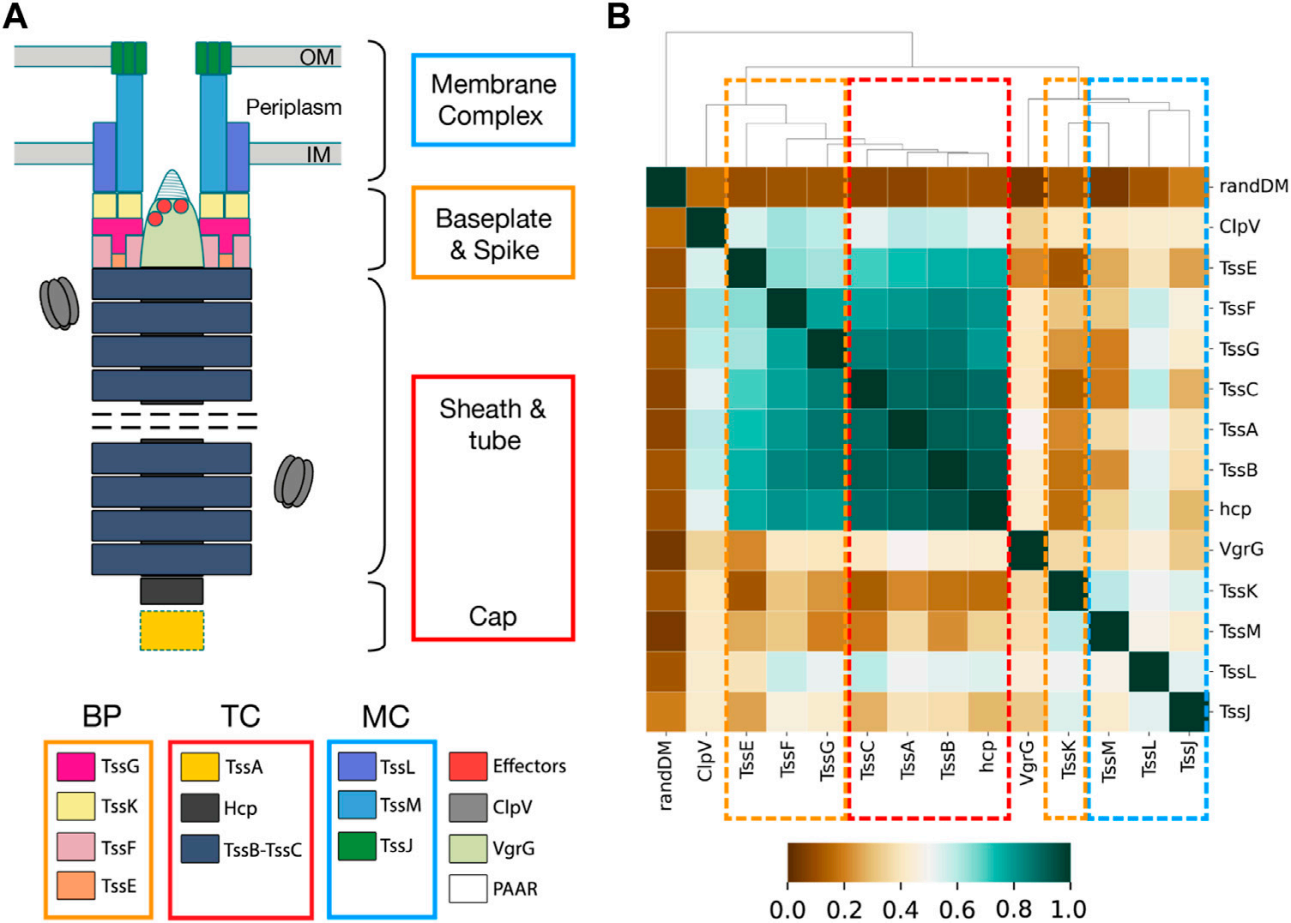
T6SS subcomplex structure and AlignScape correlation matrix for T6SS core components. **(A)** Schematic representation of T6SS structure with the TC, BP, and MC proteins highlighted with red, orange, and blue squares, respectively. **(B)** T6SS AlignScape correlation matrix depicting the correlations between all T6SS core components. randDM corresponds to a randomly generated distance matrix used as a reference of uncorrelation. TC, MC, and BP clusters of correlated genes are highlighted with red, blue, and orange dashed squares, respectively.

The U-matrix and corresponding distance matrix for the remaining T6SS^i^ core components reveal two distinct patterns. On the one hand, TssC (Supplementary Figures S6A–B), Hcp, TssG, TssE, TssF, and TssA exhibited properties similar to TssB: U-matrices organize into basins containing homogeneous subtypes, and the distance matrix dendrograms exhibit the early bifurcation of the i1-i2 and i3-i4a-i4b-i5 clades. On the other hand, TssM (Supplementary Figures S6C–D), TssJ, TssK, TssL, and VgrG do not present a clear separation of subtypes in different basins, and their dendrograms lack the early bifurcation. These two distinct patterns prompted us to analyze the correlation of TssB, TssC, and TssM distance matrices, which we interpret as a measure of gene coevolution. The correlation between TssB and TssC was 0.921, indicating strong coevolution between these 2 TC genes. In contrast, the correlation between TssB and TssM was 0.286, indicating weak coevolution. Subsequently, we extended the analysis to all T6SS^i^ core genes (Figure 6B). The resulting AlignScape correlation matrix reveals two clusters, one containing all T6SS TC genes and another with all T6SS MC genes. Remarkably, the BP genes fall between the TC and the MC clusters. TssE correlated with the TC genes but not with the MC genes. TssG and TssF showed strong correlations with the TC genes and weaker correlations with the MC genes. In contrast, TssK correlated with the MC genes but not with the TC genes. TssA and ClpV correlate with TC genes and TssG, TssF, and TssE BP genes, with TssA presenting stronger correlations overall than ClpV. Finally, VgrG did not demonstrate any correlation with the T6SS components.

To compare AlignScape with other gene coevolution methods (Pazos and Valencia, 2001; Pazos et al., 2008; de Juan et al., 2013), we calculated two additional correlation matrices: a BLOSUM62 correlation matrix (B62) and a phylogenetic correlation matrix (PHY). The B62 employed the BLOSUM62 distance (Henikoff and Henikoff, 1992) between pairs of aligned sequences, while the PHY utilized the distance extracted from phylogenetic gene trees (See Supplementary Methods). Excluding randDM, which is completely uncorrelated with all T6SS^i^ core components in all correlation matrices, both the B62 and PHY correlation matrices demonstrated a narrower dynamic range than the AlignScape correlation matrix (Supplementary Figure S7), which hinders the identification of clusters of coevolving genes.

## 4 Discussion

We presented AlignScape, a method that applies Self-Organizing Maps (SOM) on aligned sequences. SOM preserves and represents the neighboring properties of the input data in a periodic 2D grid output space. This characteristic was crucial for effectively displaying and analyzing thousands of sequences within a similarity landscape. AlignScape is depicted by a U-matrix, a periodic 3D graphical representation that captures the complexity of the high-dimensional space of an MSA. In this representation, similar sequences are enforced to be proximal within a densely filled space that incorporates the input data and its interpolation, in contrast with SSN, which are intrinsically sparse. This unique feature of AlignScape allows the creation of a continuous and intuitive similarity landscape that augments the input sequences to fully explain the MSA.

AlignScape presents several additional advantages and novelties: 1) Its U-matrix representation handles large sets of sequences, which can be challenging to visualize using standard phylogenetic trees and SSNs. This capability stems from AlignScape’s ability to coarse-grain input data, where similar sequences are located in close proximity. This significantly simplifies the visualization of extensive sequence datasets (Supplementary Figure S9). 2) The U-matrix distance between two mapped sequences defines a novel sequence distance. 3) The U-matrix distance facilitates the calculation of distance matrices and the subsequent construction of correlation matrices based on taxonomical pairing, serving as a valuable tool for studying gene coevolution. 4) Users have the flexibility to determine the level of coarse-graining of the representation. They can also infer the sequences of U-matrix units and obtain mutation pathways between input sequences. 5) Similar to SSNs and phylogenetic trees, AlignScape supports sequence annotation with external attributes, enabling inference of functional and evolutionary traits for non-annotated sequences. 6) The MST provides insights into the relationships between different regions of the U-matrix. Unlike rooted evolution trees, where the root is enforced, the MST is completely unsupervised. Additionally, the MST can be utilized to generate aperiodic U-matrices, which provide a representation that ensures topographical connection and integrity of the spatial information. 7) AlignScape is highly efficient as it does not rely on pairwise distance calculation.

We applied AlignScape to three distinct cases: the human Kinome, the human GPCRs, and the proteins from the bacterial T6SS. In each test case, the resulting U-matrices successfully arranged sequences belonging to the same functional group or phylogenetic subtype together.

In the case of the human Kinome, which had the most validated and complete MSA, the MST and the phylogenetic tree exhibited consistent connections among the kinome groups. This agreement was further supported by the similar topology between the unrooted tree and the MST. The subsequent group inference of unclassified kinases highlighted the arrangement of kinases into groups and the interconnections between these. However, certain inferences were ambiguous, primarily due to the location of unclassified kinases at the boundaries between groups and in barriers of distant sequences.

The human GPCR MSA also underwent extensive validation; however, it was limited to a protein domain, which may only capture some of the evolutionary information related to the GPCR groups and subgroups. Furthermore, the rooted phylogenetic tree makes a direct comparison with the MST challenging. Despite these challenges, the interconnections among GPCR groups in the MST generally mirrored those observed in the tree. Nonetheless, both methods face difficulties when arranging the GPCR A subgroups. AlignScape improved this situation by inferring the subgroup of unclassified A GPCRs, revealing four distinct branches within the A main branch, each representing a different A subgroup with only a few ambiguous inferences.

In the case of T6SS, our analysis began with the TssB gene. Unlike the human Kinome and GPCRs, most TssB input sequences were unclassified. Additionally, we lacked a structurally validated MSA and an accurate phylogenetic tree. Despite these limitations, and thanks to the AlignScape inference, we could replicate the T6SS^i^ subtyping and reveal groups of unclassified sequences not present in the current classification. To further investigate the relationships among T6SS^i^ subtypes, we computed the TssB distance matrix and corresponding dendrogram. Remarkably, we observed a consensus between the earliest phylogenetic trees documented in the literature (Boyer et al., 2009; Barret et al., 2013a) and the dendrogram. Both outlined the subdivision of T6SS^i^ subtypes into two branches: i1-i2 and i3-i4a-i4b-i5. AlignScape was also used to infer gene coevolution. It predicted the coevolution between TssB and TssC, two proteins originating from a common ancestor (Kudryashev et al., 2015). Instead, TssB and TssM, proteins located in distinct subcomplexes and with homologies on different bacterial systems (Durand et al., 2012; Kudryashev et al., 2015), were predicted as not coevolving. Then, the T6SS correlation matrix revealed two clusters of coevolving genes: one with TC and another with MC genes. BP genes with structural interactions with MC proteins (Vanlioğlu et al., 2023) coevolved with the MC genes but not with TC genes. Conversely, BP genes with common origin with TC genes (Cherrak et al., 2018) coevolved with them but not with MC genes. Interestingly, VgrG was found to be the least coevolving gene. These could be link to the fact that a T6SS might utilize multiple VgrG genes to translocate different substrates (Hachani et al., 2011; Zheng et al., 2011; Cianfanelli et al., 2016; Liang et al., 2021) and that these are not necessarily confined within the T6SS gene cluster (Hachani et al., 2011; Zheng et al., 2011); rather, they are mobile genetic elements. Hence, the low coevolution of VgrG could be attributed to its inherent mobile nature. In summary, the T6SS AlignScape correlation matrix provides a first quantitative assessment of the T6SS mosaic evolution, where multiple functional blocks were acquired from different bacterial machines up to the formation of the complete system. We could detect the distinctive imprint of this co-option event that likely occurred between a preexisting secretion system and the phage tail, a process hypothesized to be responsible for the formation of the T6SS complex (Denise et al., 2020).

AlignScape currently relies on precomputed MSA. Aligning sequences on the fly would enhance AlignScape robustness, especially when dealing with outlier sequences. Another AlignScape limitation is that the final U-matrix orientation is unpredictable due to the random initialization and the shuffling of the training data, making the comparison between variants of the MSA difficult. To address this issue, a method for aligning U-matrices should be developed, facilitating U-matrices comparison and streamlining gene correlation calculation without the necessity of taxonomical pairing. Finally, the data derived from the interpolation and mutation inference along pathways connecting input sequences offer the potential for computing coevolutionary patterns between sequences.

## Data Availability

AlignScape is available as a Python library (https://github.com/bougui505/alignscape). Users can utilize it locally as a Jupyter Notebook or within a Google Colaboratory Notebook (https://colab.research.google.com/github/bougui505/alignscape/blob/master/alignscape.ipynb), supporting both CPUs and GPUs. Within the notebook, we provide step-by-step instructions on how to install the necessary libraries, load the MSA, execute the SOM training, and generate several U-matrix representations. We also deployed an Apptainer (formerly known as Singularity) version of AlignScape (https://zenodo.org/records/10417520). We offer convenient downloads of all relevant files to ensure comprehensive access to the generated data. These include the resulting AlignScape object, the MST, the sequence to BMU to cluster assignment file, and images of all generated U-matrices.

## References

[B1] Ahmad N. Alahakoon D. Chau R. (2008). Classification of protein sequences using the growing self-organizing map. Int. Conf. Inf. Automation Sustain. 2008, 167–172. 10.1109/ICIAFS.2008.4783969

[B2] Alhosaini K. Azhar A. Alonazi A. Al-Zoghaibi F. (2021). GPCRs: the most promiscuous druggable receptor of the mankind. Saudi Pharm. J. SPJ 29, 539–551. 10.1016/j.jsps.2021.04.015 34194261 PMC8233523

[B3] Allsopp L. P. Bernal P. Nolan L. M. Filloux A. (2020). Causalities of war: the connection between type VI secretion system and microbiota. Cell. Microbiol. 22, e13153. 10.1111/cmi.13153 31872954 PMC7540082

[B4] Altschul S. F. Gish W. Miller W. Myers E. W. Lipman D. J. (1990). Basic local alignment search tool. J. Mol. Biol. 215, 403–410. 10.1016/S0022-2836(05)80360-2 2231712

[B5] Atkinson H. J. Morris J. H. Ferrin T. E. Babbitt P. C. (2009). Using sequence similarity networks for visualization of relationships across diverse protein superfamilies. PLOS ONE 4, e4345. 10.1371/journal.pone.0004345 19190775 PMC2631154

[B6] Barret M. Egan F. O’Gara F. (2013a). Distribution and diversity of bacterial secretion systems across metagenomic datasets. Environ. Microbiol. Rep. 5, 117–126. 10.1111/j.1758-2229.2012.00394.x 23757140

[B7] Berginski M. E. Moret N. Liu C. Goldfarb D. Sorger P. K. Gomez S. M. (2021). The Dark Kinase Knowledgebase: an online compendium of knowledge and experimental results of understudied kinases. Nucleic Acids Res. 49, D529–D535. 10.1093/nar/gkaa853 33079988 PMC7778917

[B8] Boyer F. Fichant G. Berthod J. Vandenbrouck Y. Attree I. (2009). Dissecting the bacterial type VI secretion system by a genome wide *in silico* analysis: what can be learned from available microbial genomic resources? BMC Genomics 10, 104. 10.1186/1471-2164-10-104 19284603 PMC2660368

[B9] Cherrak Y. Rapisarda C. Pellarin R. Bouvier G. Bardiaux B. Allain F. (2018). Biogenesis and structure of a type VI secretion baseplate. Nat. Microbiol. 3, 1404–1416. 10.1038/s41564-018-0260-1 30323254

[B10] Chiu J. K. H. Ong R. T.-H. (2022). Clustering biological sequences with dynamic sequence similarity threshold. BMC Bioinforma. 23, 108. 10.1186/s12859-022-04643-9 PMC8969259 35354426

[B11] Cianfanelli F. R. Diniz J. A. Guo M. Cesare V. D. Trost M. Coulthurst S. J. (2016). VgrG and PAAR proteins define distinct versions of a functional type VI secretion system. PLOS Pathog. 12, e1005735. 10.1371/journal.ppat.1005735 27352036 PMC4924876

[B12] Copp J. N. Akiva E. Babbitt P. C. Tokuriki N. (2018). Revealing unexplored sequence-function space using sequence similarity networks. Biochemistry 57, 4651–4662. 10.1021/acs.biochem.8b00473 30052428

[B13] Crisan C. V. Hammer B. K. (2020). The <i>*Vibrio cholerae*</i> type VI secretion system: toxins, regulators and consequences. Environ. Microbiol. 22, 4112–4122. 10.1111/1462-2920.14976 32133757

[B14] Cvicek V. Goddard W. A. Abrol R. (2016). Structure-based sequence alignment of the transmembrane domains of all human GPCRs: phylogenetic, structural and functional implications. PLoS Comput. Biol. 12, e1004805. 10.1371/journal.pcbi.1004805 27028541 PMC4814114

[B15] de Juan D. Pazos F. Valencia A. (2013). Emerging methods in protein co-evolution. Nat. Rev. Genet. 14, 249–261. 10.1038/nrg3414 23458856

[B16] Denise R. Abby S. S. Rocha E. P. C. (2020). The evolution of protein secretion systems by Co-option and tinkering of cellular machineries. Trends Microbiol. 28, 372–386. 10.1016/j.tim.2020.01.005 32298615

[B17] Durand E. Zoued A. Spinelli S. Watson P. J. H. Aschtgen M.-S. Journet L. (2012). Structural characterization and oligomerization of the TssL protein, a component shared by bacterial type VI and type IVb secretion systems. J. Biol. Chem. 287, 14157–14168. 10.1074/jbc.M111.338731 22371492 PMC3340138

[B18] Edgar R. C. (2010). Search and clustering orders of magnitude faster than BLAST. Bioinforma. Oxf. Engl. 26, 2460–2461. 10.1093/bioinformatics/btq461 20709691

[B19] Emes R. D. (2008). Inferring function from homology. Methods Mol. Biol. Clifton N. J. 453, 149–168. 10.1007/978-1-60327-429-6_6 18712301

[B20] Ferrán E. A. Ferrara P. (1991). Topological maps of protein sequences. Biol. Cybern. 65, 451–458. 10.1007/BF00204658 1958730

[B21] Ferrán E. A. Pflugfelder B. Ferrara P. (1994). Self-organized neural maps of human protein sequences. Protein Sci. Publ. Protein Soc. 3, 507–521. 10.1002/pro.5560030316 PMC2142706 8019421

[B22] Fredriksson R. Lagerström M. C. Lundin L.-G. Schiöth H. B. (2003). The G-protein-coupled receptors in the human genome form five main families. Phylogenetic analysis, paralogon groups, and fingerprints. Mol. Pharmacol. 63, 1256–1272. 10.1124/mol.63.6.1256 12761335

[B23] Fu L. Niu B. Zhu Z. Wu S. Li W. (2012). CD-HIT: accelerated for clustering the next-generation sequencing data. Bioinformatics 28, 3150–3152. 10.1093/bioinformatics/bts565 23060610 PMC3516142

[B24] Hachani A. Lossi N. S. Hamilton A. Jones C. Bleves S. Albesa-Jové D. (2011). Type VI secretion system in *Pseudomonas aeruginosa*: secretion and multimerization of VgrG proteins. J. Biol. Chem. 286, 12317–12327. 10.1074/jbc.M110.193045 21325275 PMC3069435

[B25] Henikoff S. Henikoff J. G. (1992). Amino acid substitution matrices from protein blocks. Proc. Natl. Acad. Sci. 89, 10915–10919. 10.1073/pnas.89.22.10915 1438297 PMC50453

[B26] Jana B. Salomon D. (2019). Type VI secretion system: a modular toolkit for bacterial dominance. Future Microbiol. 14, 1451–1463. 10.2217/fmb-2019-0194 31718312

[B27] Jumper J. Evans R. Pritzel A. Green T. Figurnov M. Ronneberger O. (2021). Highly accurate protein structure prediction with AlphaFold. Nature 596, 583–589. 10.1038/s41586-021-03819-2 34265844 PMC8371605

[B28] Kohonen T. (1982). Self-organized formation of topologically correct feature maps. Biol. Cybern. 43, 59–69. 10.1007/BF00337288

[B29] Krishnan A. Almén M. S. Fredriksson R. Schiöth H. B. (2012). The origin of GPCRs: identification of mammalian like rhodopsin, adhesion, glutamate and frizzled GPCRs in fungi. PLOS ONE 7, e29817. 10.1371/journal.pone.0029817 22238661 PMC3251606

[B30] Kudryashev M. Wang R. Y.-R. Brackmann M. Scherer S. Maier T. Baker D. (2015). Structure of the type VI secretion system contractile sheath. Cell. 160, 952–962. 10.1016/j.cell.2015.01.037 25723169 PMC4359589

[B31] Lagerström M. C. Schiöth H. B. (2008). Structural diversity of G protein-coupled receptors and significance for drug discovery. Nat. Rev. Drug Discov. 7, 339–357. 10.1038/nrd2518 18382464

[B32] Li W. Jaroszewski L. Godzik A. (2001). Clustering of highly homologous sequences to reduce the size of large protein databases. Bioinformatics 17, 282–283. 10.1093/bioinformatics/17.3.282 11294794

[B33] Liang X. Pei T.-T. Li H. Zheng H.-Y. Luo H. Cui Y. (2021). VgrG-dependent effectors and chaperones modulate the assembly of the type VI secretion system. PLOS Pathog. 17, e1010116. 10.1371/journal.ppat.1010116 34852023 PMC8668125

[B34] Manning G. Whyte D. B. Martinez R. Hunter T. Sudarsanam S. (2002). The protein kinase complement of the human genome. Science 298, 1912–1934. 10.1126/science.1075762 12471243

[B35] Modi V. Dunbrack R. L. (2019). A structurally-validated multiple sequence alignment of 497 human protein kinase domains. Sci. Rep. 9, 19790. 10.1038/s41598-019-56499-4 31875044 PMC6930252

[B36] NCBI News (2023). Spring 2004|BLASTLab. Available at: https://www.ncbi.nlm.nih.gov/Web/Newsltr/Spring04/blastlab.html (Accessed July 6, 2023).

[B37] Pazos F. Juan D. Izarzugaza J. M. G. Leon E. Valencia A. (2008). Prediction of protein interaction based on similarity of phylogenetic trees. Methods Mol. Biol. Clifton N. J. 484, 523–535. 10.1007/978-1-59745-398-1_31 18592199

[B38] Pazos F. Valencia A. (2001). Similarity of phylogenetic trees as indicator of protein-protein interaction. Protein Eng. 14, 609–614. 10.1093/protein/14.9.609 11707606

[B39] Sana T. G. Baumann C. Merdes A. Soscia C. Rattei T. Hachani A. (2015). Internalization of *Pseudomonas aeruginosa* strain PAO1 into epithelial cells is promoted by interaction of a T6SS effector with the microtubule network. mBio 6, e00712. 10.1128/mBio.00712-15 26037124 PMC4453011

[B40] Sanderson T. Bileschi M. L. Belanger D. Colwell L. J. (2023). ProteInfer, deep neural networks for protein functional inference. eLife 12, e80942. 10.7554/eLife.80942 36847334 PMC10063232

[B41] Shannon P. Markiel A. Ozier O. Baliga N. S. Wang J. T. Ramage D. (2003). Cytoscape: a software environment for integrated models of biomolecular interaction networks. Genome Res. 13, 2498–2504. 10.1101/gr.1239303 14597658 PMC403769

[B42] Sharman J. L. Benson H. E. Pawson A. J. Lukito V. Mpamhanga C. P. Bombail V. (2013). IUPHAR-DB: updated database content and new features. Nucleic Acids Res. 41, D1083–D1088. 10.1093/nar/gks960 23087376 PMC3531077

[B43] Steinegger M. Meier M. Mirdita M. Vöhringer H. Haunsberger S. J. Söding J. (2019). HH-suite3 for fast remote homology detection and deep protein annotation. BMC Bioinforma. 20, 473. 10.1186/s12859-019-3019-7 PMC6744700 31521110

[B44] Taylor N. M. I. van Raaij M. J. Leiman P. G. (2018). Contractile injection systems of bacteriophages and related systems. Mol. Microbiol. 108, 6–15. 10.1111/mmi.13921 29405518

[B45] Vanlioğlu E. Santin Y. G. Filella-Merce I. Pellarin R. Cascales E. (2023). Coevolution-guided mapping of the type VI secretion membrane complex-baseplate interface. J. Mol. Biol. 435, 167918. 10.1016/j.jmb.2022.167918 36509161

[B46] Wei D. Jiang Q. Wei Y. Wang S. (2012). A novel hierarchical clustering algorithm for gene sequences. BMC Bioinforma. 13, 174. 10.1186/1471-2105-13-174 PMC3443659 22823405

[B47] Yang D. Zhou Q. Labroska V. Qin S. Darbalaei S. Wu Y. (2021). G protein-coupled receptors: structure- and function-based drug discovery. Signal Transduct. Target. Ther. 6, 7. 10.1038/s41392-020-00435-w 33414387 PMC7790836

[B48] Yoon B.-J. (2009). Hidden Markov models and their applications in biological sequence analysis. Curr. Genomics 10, 402–415. 10.2174/138920209789177575 20190955 PMC2766791

[B49] Zalewska M. Siara M. Sajewicz W. (2014). G protein-coupled receptors: abnormalities in signal transmission, disease states and pharmacotherapy. Acta Pol. Pharm. 71, 229–243. 25272642

[B50] Zheng J. Ho B. Mekalanos J. J. (2011). Genetic analysis of anti-amoebae and anti-bacterial activities of the type VI secretion system in *Vibrio cholerae* . PLOS ONE 6, e23876. 10.1371/journal.pone.0023876 21909372 PMC3166118

