# Supplementary material for: AlignScape, displaying sequence similarity using self-organizing maps
Source: Front Bioinform. 2024 Jan 26;4:1321508. doi: 10.3389/fbinf.2024.1321508 (PMC10853471; doi:10.3389/fbinf.2024.1321508)
Supplement: Supplementary file 1 [file DataSheet1.pdf]

AlignScape, displaying sequence similarity using self-organizing maps

Supplementary Material

1 Supplementary Methods

1.1 Position probability matrix (PPM) representation

Given a set of p aligned sequences of length l, its PPM M is a matrix with l rows and 25 columns. Each element m_{ij} of M represents the frequency of the *jth* amino acid symbol from the list "ABCDEFGHIKLMNPQRSTVWXYZ|-" at the *ith* site of the alignment. Apart from the symbol of the 20 naturally occurring amino acids, the list includes B (Aspartic acid or Asparagine), Z (Glutamic acid or Glutamine), X (any amino acid), '-' (gap extension), and '|' (gap opening). The PPM of a single sequence is a one-hot encoded matrix, meaning it is a sparse matrix filled with 0 everywhere except for the element m_{ij} = 1 whose *jth* symbol corresponds to the *ith* amino acid of the sequence.

1.2 Position probability matrix distance (PPMd)

The distance between PPMs is based on the ScoreDist distance estimator (Sonnhammer and Hollich, 2005) and the closeness metric described in (Cherrak et al., 2021). Given two PPMs M_a and M_b of dimension $l \times 25$ and elements a_{ij} and b_{ij}, respectively, we defined its distance (PPMd) as:

$$PPMd(M_a, M_b) = -ln(\frac{S(M_a, M_b) - S_R(M_a, M_b)}{S_I(M_a, M_b) - S_R(M_a, M_b)}) \cdot 100.$$

$S(M_a, M_b)$ is the BLOSUM62 based score between M_a and M_b:

$$\begin{aligned} S(M_a, M_b) &= \mathrm{tr}(M_a^* \cdot B_{62} \cdot (M_b^*)^T) + \textstyle\sum_i^l(\max(a_{i|}, b_{i|})) \cdot G_o + \\ &+ \textstyle\sum_i^l(\max(a_{i-}, b_{i-})) \cdot G_e \end{aligned},$$

where M_a^* and M_b^* are $l \times 23$ matrices obtained by removing the gap opening and gap extension columns from M_a and M_b, respectively; B_{62} is the 23×23 BLOSUM62 scoring matrix; *tr* is the trace operation; $a_{i|}$ and $b_{i|}$ are the gap opening frequencies at the *ith* site of M_a and M_b, respectively; a_{i-} and b_{i-} are the gap extension frequencies at the *ith* site of M_a and M_b, respectively; G_o is the gap opening penalty and G_e the gap extension penalty. $S_R(M_a, M_b)$ is defined as the expected score between two unrelated PPM:

$$S_R(M_a, M_b) = l \cdot \frac{\sum_i^m \sum_j^m c_{ij} + m \cdot (G_o + G_e)}{2m + m^2},$$

where c_{ij} are the BLOSUM62 elements (m=23 is the BLOSUM62 square matrix dimension). Thus, $S_R(M_a, M_b)$ can be considered as the lower limit of the score between M_a and M_b. Importantly, if M_a and M_b are very distant or G_o and G_e are set too low, it might happen that $S(M_a, M_b) < S_R(M_a, M_b)$. In such a case, $S(M_a, M_b)-S_R(M_a, M_b)$ is set heuristically to a minimum value of 0.001. On the other hand, the upper limit of the score between M_a and M_b is the score obtained when the two PPM are identical, i.e., $S_I(M_a, M_b)$:

$$S_I(M_a, M_b) = \frac{S(M_a, M_a) + S(M_b, M_b)}{2}$$

As PPMd can be expressed through vectorial algebra, it was implemented in a pytorch call to perform calculations on GPU.

1.3 MSA clustering with a self-organizing map (AlignScape)

AlignScape is an adaptation of a code where SOM was developed to analyze the conformational space of protein MD trajectories (Bouvier et al., 2015; Mallet et al., 2021). AlignScape takes an MSA as input and utilizes the PPMd as a metric. To avoid the border effects of a planar output space (Mount and Weaver, 2011), AlignScape sets the output SOM as a $n \times n$ toroidal map where each unit is initialized as a random PPM computed using a uniform distribution U(0,1). Hence, AlignScape reduces an input MSA of $l{\cdot}25$ variables (where l is the alignment length and 25 all different alignment symbols) and p observations (total number of aligned sequences) to a n^2 output SOM.

AlignScape training runs consecutive training cycles named epochs. During an epoch, each MSA sequence is iteratively sampled once. When selected, each aligned sequence is converted into PPM and compared against all SOM units to identify its BMU. Then, the PPMs of the BMU and its neighboring units are updated to resemble the sampled sequence. The function that regulates the neighbor selection radius (σ) is a Gaussian, which decays exponentially with the iterations, from a fourth of the SOM dimension (n/4) to 1. The learning rate (α), which controls how much we modify the PPMs of the BMU and its neighbors, decreases exponentially from 0.5 to 0. To maximize efficiency, each iteration can be structured in batches of sequences rather than in individual sequences.

AlignScape training can be performed on both CPUs and GPUs. Our CPU implementation was developed based on Bouvier et al. work (Bouvier et al., 2015) and our GPU implementation was built upon the subsequent work by Mallet et al. (Mallet et al., 2021), where they specifically optimized their method for GPU.

1.4 U-matrix representation

As the output SOM, the U-matrix is a $n \times n$ periodic grid. More specifically, the value of each U-matrix unit is the mean PPMd between the corresponding SOM unit and its eight adjacent neighboring units. Here, the U-matrix is plotted using a heatmap, where dark-blue units represent units whose sequences are similar to the sequences of their neighboring units. In contrast, yellow units represent units whose sequences diverge from the neighbor unit sequences. Hence, regions of contiguous blue-colored units

are interpreted as clusters of similar sequences (basin) and yellow regions as borders between clusters (barrier).

Sequences from the input MSA can be mapped to the U-matrix using their respective BMUs. Subsequently, these BMUs can be color-coded according to prior information associated with their corresponding sequences. This sequence annotating process enables the identification of insightful patterns within the U-matrix.

1.5 U-matrix distance

Given two aligned sequences s_a and s_b, their corresponding PPMs M_a and M_b, and their corresponding U-matrix BMUs, BMU_a and BMU_b, we defined its U-matrix distance as the length of the shortest path between BMU_a and BMU_b. Importantly, the distance between two U-matrix adjacent units is calculated as the norm of their values. SciPy Python library (Virtanen et al., 2020) was used to compute the shortest path between non-adjacent units.

1.6 Quantization and topographical error

The quantization error (QE) is the mean PPMd between the PPMs of the input sequences and the PPMs of their corresponding BMUs. Hence, the QE is a descriptor representing the similarity between the input MSA and the trained SOM. The smaller the QE, the better SOM fits the initial data.

The topographical error (TE) is the proportion of input sequences for which its best matching unit and its second best matching unit are not adjacent. TE estimates to which extent SOM preserves the topology of the input data. The closer the TE is to 0, the better.

1.7 Robustness and scalability of AlignScape

There are several user-defined parameters needed to perform a AlignScape calculation: the learning rate (α), the neighbor selection radius (σ), the size of the map ($n \times n$), the size of the batch, and the number of epochs. The values of α and σ were empirically determined by trial and error. The σ parameter specifically affects the coarse-graining of the sequences, a smaller radius forces all sequences to be assigned to different units. The map size should be larger than the input MSA, and its maximal size is limited by the computational power (i.e., GPU or CPU memory). Similarly, the batch size is limited by the processing unit's memory and trade-off between the learning time and the map size. In our trials, we set the batch size of 10 when the map has a size of 90×90. Memory allocation depends on several parameters: the batch size, the SOM size, and the length of the MSA. Trimming the original alignment, decreasing the batch size, or reducing the SOM size can facilitate the accommodation of lengthy MSAs. On the other hand, execution time is dependent on the batch size, the SOM size, the MSA length, and the number of sequences within the MSA. AlignScape's execution time increases as the SOM size, the MSA length, and the number of MSA sequences grow. To mitigate execution time, one might consider increasing the batch size, although this must be balanced against memory allocation. Suppl. Figure 10A illustrates the increase in memory allocation as the batch size increases, while Suppl. Figure 10B showcases the average execution time for a training epoch. This

data is valuable in selecting an appropriate batch size for a specific MSA length, aiding in optimizing execution times.To evaluate the convergence of our AlignScape calculations, we employed two error measurements: quantization and topographical errors (QE and TE). We observed that the convergence was achieved after 100 epochs (Suppl. Figure 7). Different runs consistently generated similar U-matrices during this stage, even with various random initializations.

1.8 Inference of non-annotated sequences

By leveraging sequence annotation within the U-matrix, we can infer information for non-annotated sequences. To do so, we employed the k-nearest neighbors algorithm. The annotated sequences are utilized as the training set, with their corresponding BMUs serving as feature vectors and the annotated information acting as labels. The U-matrix distance is utilized as the algorithm metric. In cases where multiple labels had an equal number of neighbors during the inference stage, we implemented a procedure to resolve the tie. This procedure involves calculating the sum of distances of all k-nearest neighbors assigned to a label. The label with the lowest total distance is then selected as the final inference.

1.9 Graph-based representation of the SOM

The SOM can be represented as a square grid graph, accompanied by its corresponding adjacency matrix. In this graph, the SOM units serve as the nodes, and the connections to their eight direct neighbors in the grid are represented as edges. In addition, one can define a graph representation and its corresponding meta-adjacency matrix based on a sample of sequences $\{s_i\}$ of the MSA, where the nodes are the $\{BMU_i\}$ of these sequences and the edges are the U-matrix distances between the units.

1.10 Minimum spanning tree (MST)

The minimum spanning tree (MST) is defined as the set of shortest paths that connect $\{BMU_i\}$ corresponding to a sample of sequences $\{s_i\}$. The MST is required not to have cycles, and the path lengths are computed as U-matrix distances. To compute the MST, we utilize the sparse.csgraph.minimum_spanning_tree from Scipy Python (Virtanen et al., 2020) from the meta-adjacency matrix of $\{s_i\}$.

1.11 Aperiodic U-matrix

Given a sample of sequences $\{s_i\}$ and its MST, we can transform the periodic U-matrix into a planar aperiodic U-matrix as follows:

1. The aperiodic U-matrix is defined as a *3n x 3n* map subdivided into *3 x 3* smaller maps of size *n x n*, where *n* is the dimension of the SOM.
2. Iteratively, starting from the closest pair of connected BMUs of the MST, all pairs (BMU_1, BMU_2) with coordinates (x_1, y_1) and (x_2, y_2) are remapped into the aperiodic U-matrix as follows: i) BMU_1 is remapped into the central smaller map of the aperiodic U-matrix with coordinates $rBMU_1 = (x_1 + n, y_1 + n)$). ii) The coordinates of the BMU_2 into the nine smaller maps of the aperiodic U-matrix are calculated ((x_2, y_2), ($x_2 + n$, y_2), ($x_2 + 2n$, y_2), (x_2, $y_2 + n$), ($x_2 + n$, $y_2 + n$), ($x_2 + 2n$, $y_2 + n$), (x_2, $y_2 + 2n$), ($x_2 + n$, $y_2 + 2n$) and ($x_2 + 2n$, $y_2 + 2n$)). The

BMU_2 coordinates with the lowest Euclidean distance against $rBMU_1$ are set as $rBMU_2$. iii) The aperiodic U-matrix value of $rBMU_1$ and $rBMU_2$ units are set as the value of BMU_1 and BMU_2, respectively. While iterating over the BMUs pairs, it could happen that either BMU_1 or BMU_2 has already been remapped. In this scenario, the coordinates of the already remapped BMU are kept, and the coordinates of its partner are calculated as specified in ii) onwards.

3. The periodic U-matrix units $\{BMU_j\}$ not included in $\{BMU_i\}$ are remapped into the aperiodic U-matrix as follows: i) the BMU_i from $\{BMU_i\}$ with the smallest U-matrix distance against BMU_j is calculated ii) the coordinates of BMU_j into the nine smaller maps are calculated, and the ones with the lowest Euclidean distance against $rBMU_i$ is set as the $rBMU_j$ iii) the value of $rBMU_j$ is set as the value of BMU_j.
4. The values of the units of the aperiodic U-matrix without an associated periodic U-matrix unit are set as infinite.

1.12 Clustering

To cluster the U-matrix units, we used an Agglomerative Clustering algorithm. As a clustering metric, we employed the U-matrix distance. The number of clusters for the Agglomerative algorithm was set as the total number of local minima in the U-matrix. To calculate these minima, we utilized the U-matrix unit values and enforced a minimal Euclidean distance of seven units between each minima. The clustering algorithm was performed by cluster.AgglomerativeClustering from scikit-learn Python (Pedregosa et al., 2011) and the count of local minima by skimage.feature.peak_local_max from scikit-image (Walt et al., 2014). In the latter, the U-matrix unit values were multiplied by -1 to locate the local minimums instead of the local maximums.

1.13 AlignScape distance matrices

Given a sample of k sequences $\{s_i\}$ and their corresponding $\{BMU_i\}$, we defined its AlignScape distance matrix as a k x k matrix with elements d_{ij} corresponding to the U-matrix distance between the *ith* and the *jth* sequences. All AlignScape distance matrices were hierarchically clustered using the Seaborn Python library (Waskom et al., 2017).

1.14 Linear correlation coefficient between AlignScape distance matrices

A sequence s_A and a sequence s_B mapped in U_A and U_B U-matrices, respectively, are defined as a duplet if they have a common genetic origin (gene, gene cluster, …). Given a_i and b_i distances from D_A and D_B AlignScape distance matrices, respectively, they are defined as correlated distances if they represent a distance between the same two duplets (Suppl. Figure 1).

The Pearson correlation coefficient r between D_A and D_B is calculated as:

$$r(D_A, D_B) = \frac{\sum_i^t (a_i - \bar{a})(b_i - \bar{b})}{\sqrt{\sum_i^t (a_i - \bar{a})^2}\sqrt{\sum_i^t (b_i - \bar{b})^2}},$$

where a_i and b_i are the correlated distances between D_A and D_B, t is the total number of correlated distances, and $\bar{a}$ and $\bar{b}$ are the means of all a_i and all b_i, respectively.

Given a set {D_i} of AlignScape distance matrices, its AlignScape correlation matrix is calculated by pairwisely computing its *r*.

1.15 Phylogenetic and BLOSUM62 correlation matrices

The phylogenetic correlation matrix was calculated using gene phylogenetic distance matrices. These distance matrices were obtained by computing the gene phylogenetic trees of the sequences mapped in the AlignScape U-matrices. The phylogenetic trees were generated following the subsequent steps: i) the aligned sequences forming the phylogenetic tree were extracted from the AlignScape inputting MSA. ii) The corrected Akaike Information criteria (Akaike, 1998) calculated by Iqtree (Kalyaanamoorthy et al., 2017) was used to determine the tree best-fit maximum likelihood model (Suppl. Table 5). iii) Iqtree calculated the tree with 1000 bootstrap replicates. iv) SeaView (Gouy et al., 2010) was used to visualize the final phylogenetic tree. To extract the distances between tree leaves and generate the phylogenetic distance matrices, we utilized the Phylo package from BioPython (Talevich et al., 2012).

The BLOSUM62 correlation matrix was computed using gene BLOSUM62 distance matrices. These were calculated with the BLOSUM62 distance between the sequences mapped in the AlignScape U-matrices. BLOSUM62 distance was computed using the BioPython package.

1.16 Data gathering

1.16.1 Human Kinome

The structure-based MSA of the human Kinome containing 497 typical kinase domains was downloaded from *Modi V. et al.* (Modi and Dunbrack, 2019). The authors estimated an accuracy of 97% for this MSA by pairwisely superimposing 272 known structures of human kinases. Importantly, each MSA sequence was labeled according to its kinase group (AGC, CAMK, STE, CMGC, TKL, TK, CK1, RGC, TYR, NEK, and Other). We modified the downloaded MSA by removing the sites with low structural similarity (sites in lowercase) as well as non-informative sites (using ClipKit (ClipKIT: A multiple sequence alignment trimming software for accurate phylogenomic inference | PLOS Biology, n.d.). Additionally, we used cd-hit (Li et al., 2001) to remove redundant sequences (99% sequence identity threshold).

1.16.2 Human GPCRs

The MSA of the seven transmembrane domains of all 817 human G-protein coupled receptors (GPCRs) was downloaded from *Cvicek V et al.* (Cvicek et al., 2016). This MSA was generated and validated with experimental GPCR structures. Each aligned sequence was labeled in one of the 12 GPCR subgroups (A-α, A-β, A-γ, A-δ, A-other, olfactory, taste2, vomeronasal, B, Adeshion, C, and F) among the four major GPCR groups (A, B, C, and F). We used cd-hit to remove redundant sequences (99% sequence identity threshold) and ClipKit to remove non-informative sites from the downloaded MSA.

1.16.3 T6SS^i gene clusters

Twenty-six genomes of pathogenic proteobacteria expressing at least one T6SS^i were selected from the KEGG database (Kanehisa and Goto, 2000). From the 26 genomes, we extracted 56 T6SS gene clusters and classified them according to their subtypes (Suppl. Table 4). For each gene cluster, the corresponding TssB sequence was searched in the SecreT6 database (Li et al., 2015) (which has subtype annotation) using Blast (Madden and Camacho, 2021). The gene cluster subtype was assigned according to the subtype of the closest blast hit. To maximize the variety of T6SS^i, the genomes were chosen based on a thorough literature search.

The sequences of the 13 essential T6SS genes (TssA, TssB, TssC, TssE, TssF, TssG, TssJ, TssK, TssL, TssM, hcp, ClpV, and VgrG) were extracted from each gene cluster and used as queries on a Blast search on Uniref90 database (Suzek et al., 2015). Resulting homologous sequences whose length was shorter than 50% or longer than 150% of the length of the query were discarded. Redundant hits with an identity higher than 90% were clustered using cd-hit (Li et al., 2001).

We generated an MSA for each essential T6SS gene by iterating two steps: 1) sequence alignment of the filtered hits using MAFFT (Katoh et al., 2002) and 2) outliers removal using EvalMSA (Chiner-Oms and González-Candelas, 2016). This iterative process stops either at the 5th round or when EvalMSA could not find any outlier. Finally, we used ClipKit (ClipKIT: A multiple sequence alignment trimming software for accurate phylogenomic inference | PLOS Biology, n.d.) on the latest MSA to remove the non-informative sites.

2 Supplementary Figures and Tables

2.1 Supplementary Figures

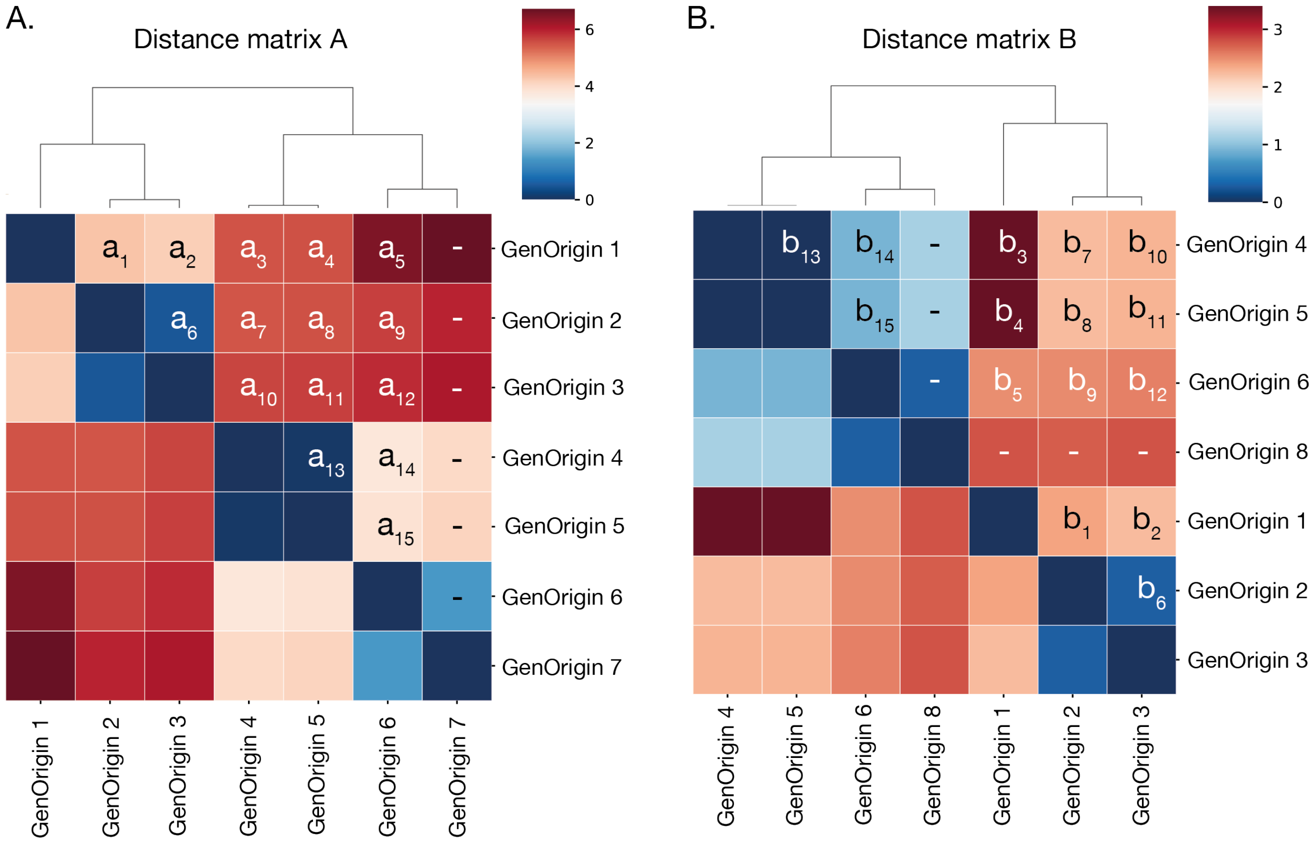

Supplementary Figure 1) Visual representation of the correlated distances between the D_A and the D_B. Subindexes of a_i distances determined the subindex of the corresponding b_i correlated distances. Dashes were utilized for non-correlated distances of D_A and D_B.

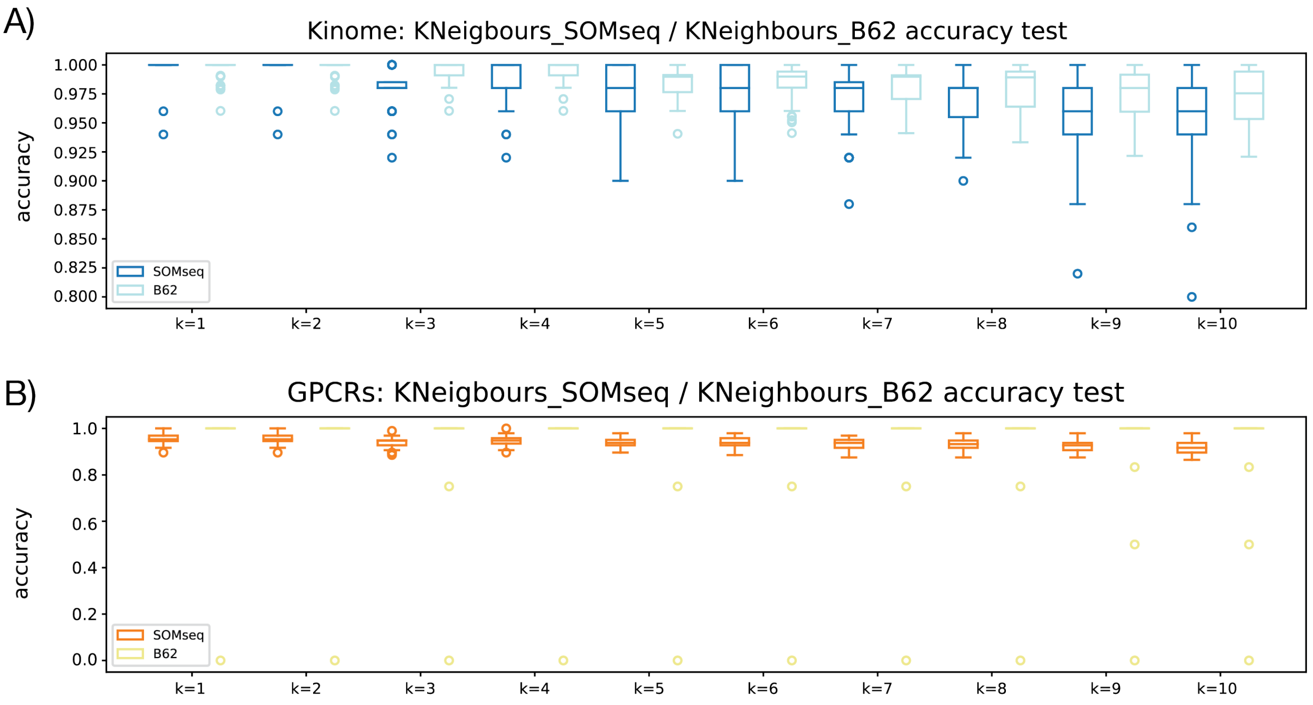

Supplementary Figure 2) Assessment of the k-neighbors inference analysis A-B) Box plots illustrating the accuracy of multiple k values of the k-neighbour algorithm after 40 inference test. At each repetition, all annotated U-matrix units were randomly divided into a training set (75%) and a testing set (25%). Then, a k-neighbor algorithm was employed to infer the group of the units in the testing set, and the accuracy was computed accordingly. For AlignScape inference (dark blue in panel A and orange in panel B), the k-neighbour algorithm utilized the U-matrix distance as a metric. On the other hand, for B62 inference (light blue in panel A and yellow in panel B), the k-neighbour algorithm employed the BLOSUM62 distance of the mapped sequences as a metric.

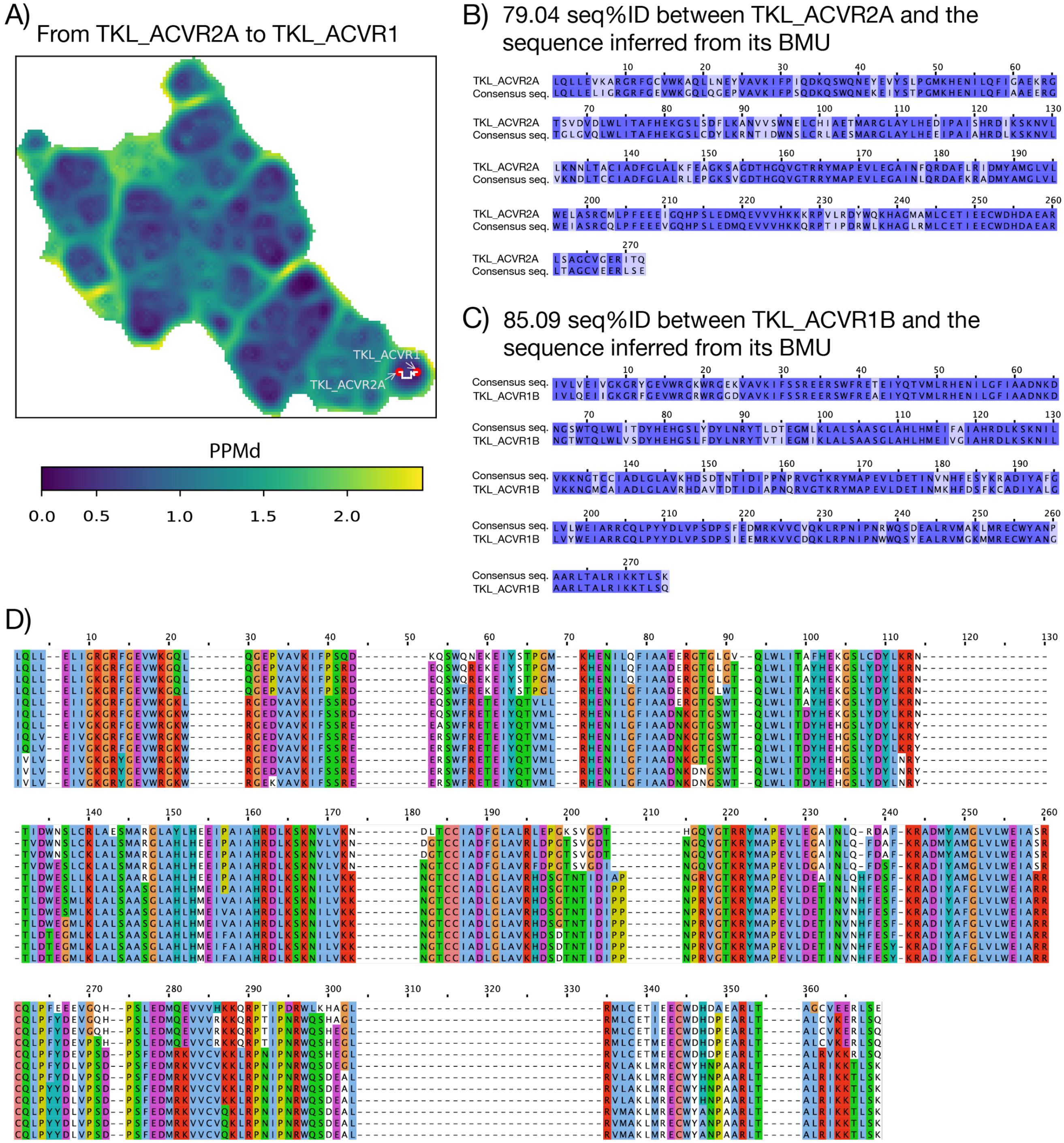

Supplementary Figure 3) Mutation pathway between two kinases from the human Kinome A) Human Kinome aperiodic U-matrix annotated with TKL_ACVR2A and TKL_ACVR1B kinases along with the shortest path connecting both **B)** Pairwise alignment and sequence percentage identity of TKL_ACVR2A and the sequence inferred from its BMU **C)** Pairwise alignment and sequence percentage identity of TKL_ACVR1B and the sequence inferred from its BMU **D)** MSA of the inferred sequences from the units belonging to the shortest path connecting TKL_ACVR2A and TKL_ACVR1B.

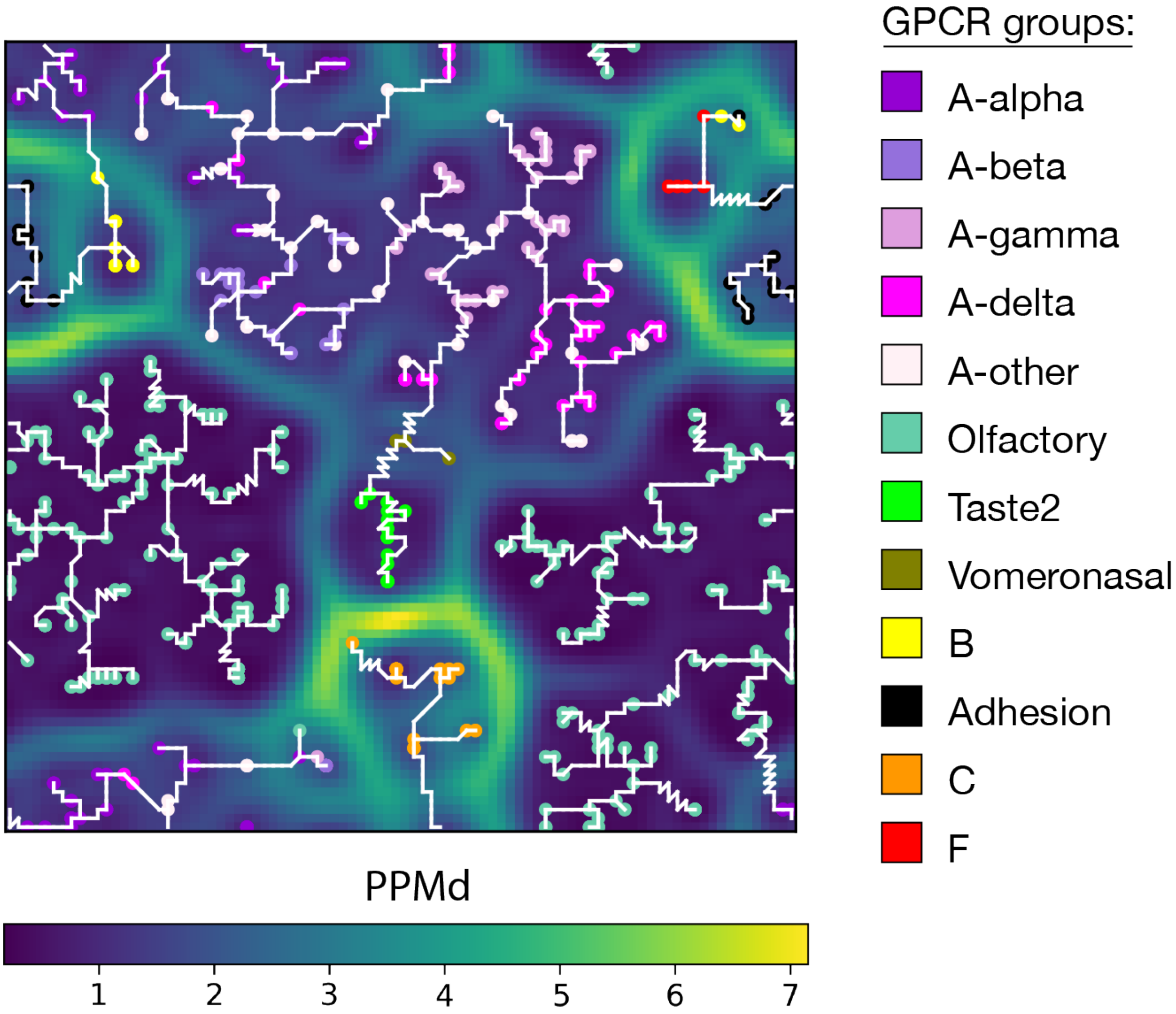

Supplementary Figure 4) GPCRs periodic U-matrix. Sequence similarity landscape of the human GPCRs represented with a periodic U-matrix. Input MSA sequences were mapped to their BMUs which were colored according to the GPCR group/subgroup of the sequences.

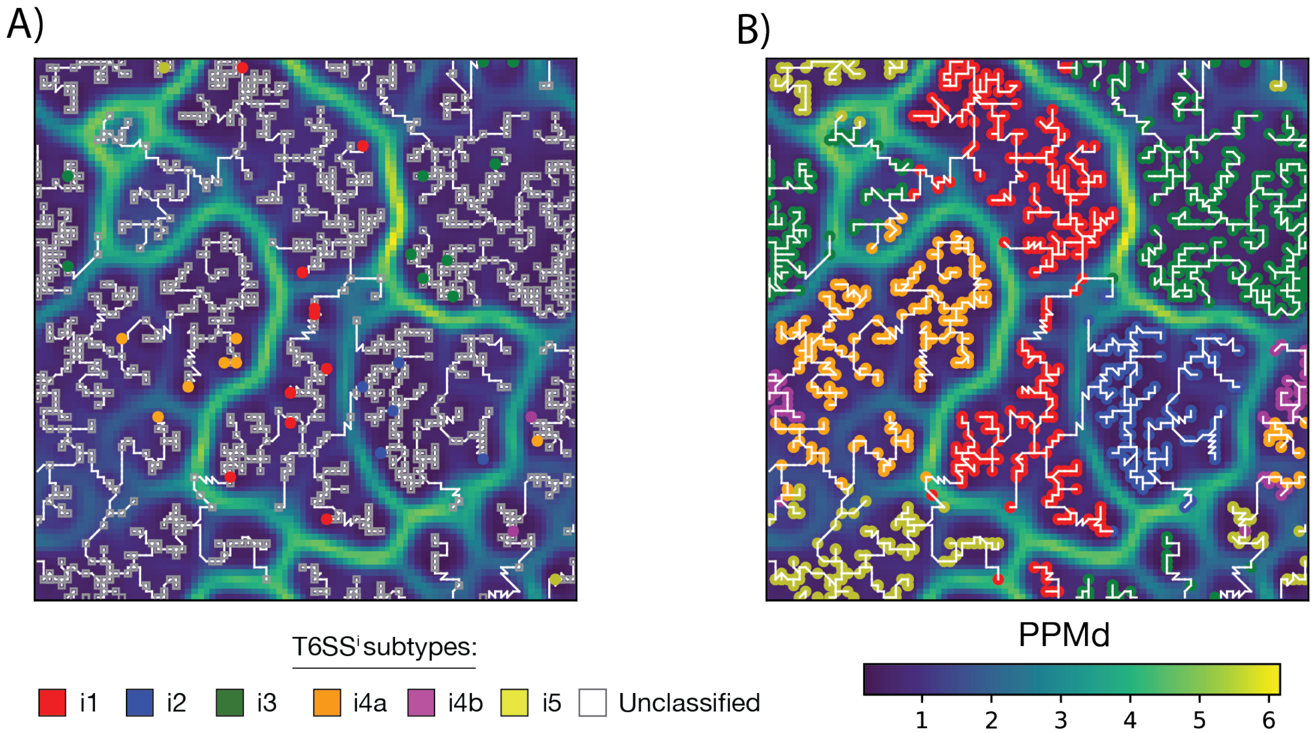

Supplementary Figure 5) TssB periodic U-matrix. A) Sequence similarity landscape of the TssB gene represented with a periodic U-matrix. Each sequence from the input MSA was mapped to its BMU which was colored according to sequence T6SS^{i} subtype. Gray squared units along the MST correspond to the BMUs of the sequences without prior classification data. **B)** U-matrix from panel A, where colored BMUs substituted the gray squared BMUs of the sequences without prior classification according to the inferred T6SS^{i} subtype. The inference was performed using a k-nearest neighbors algorithm with k=1.

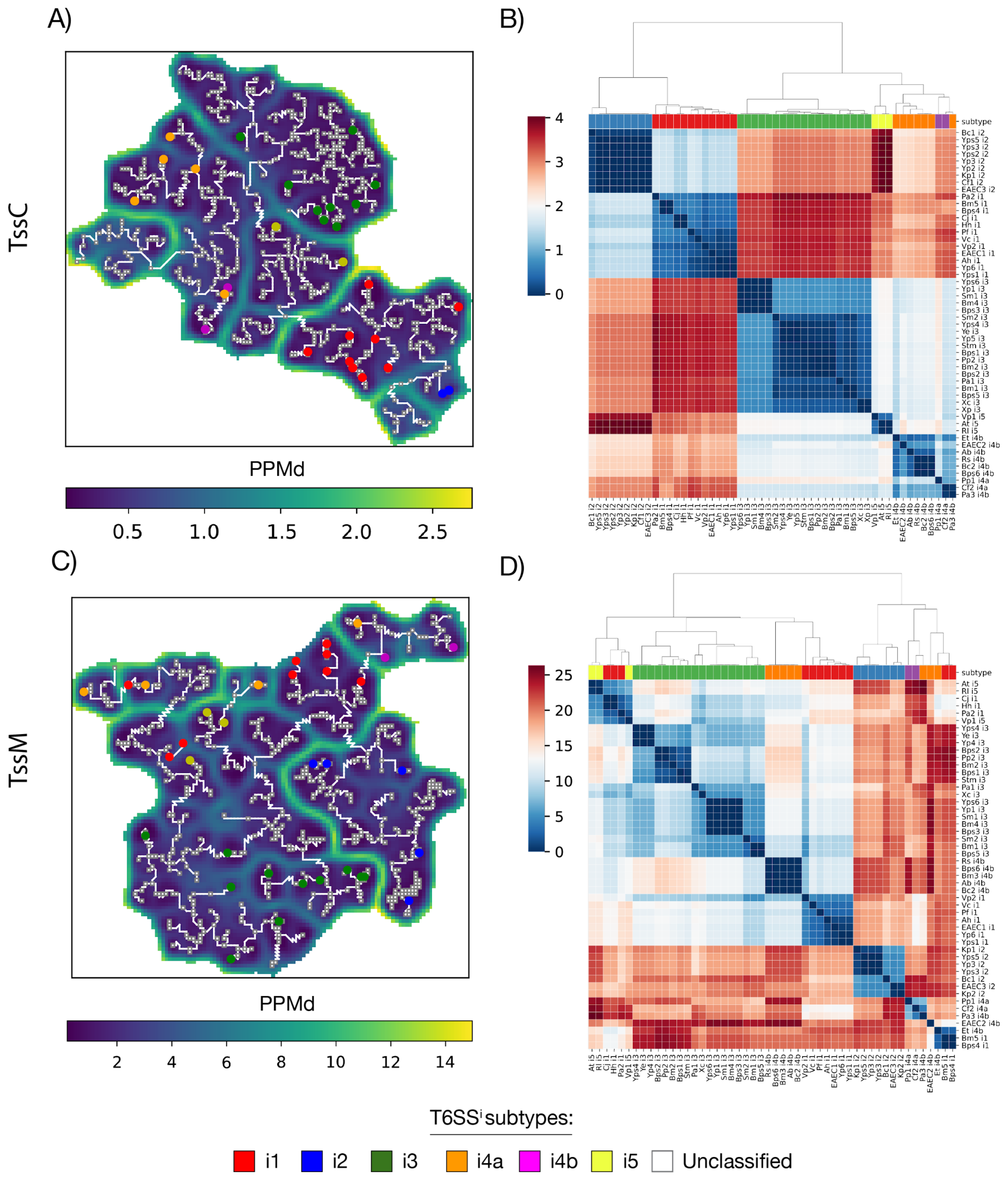

Supplementary Figure 6) TssC and TssM aperiodic U-matrices and distance matrices. In the U-matrices, each sequence from the input MSA was mapped to its BMU, which was colored according to the T6SS^i subtype of the sequence. Gray squared BMUs along the MST represent MSA sequences without prior classification data. In the distance matrices, colored boxes between the matrix and the dendrogram indicate the T6SS^i subtype of each sequence, and sequence acronyms were reported in

Suppl. Table 4. **A)** Sequence similarity landscape of the TssC gene represented with an aperiodic U-matrix. **B)** TssC distance matrix computed with sequences with prior classification data. **C)** Sequence similarity landscape of the TssM gene represented with an aperiodic U-matrix. D) TssM distance matrix computed with sequences with prior classification data.

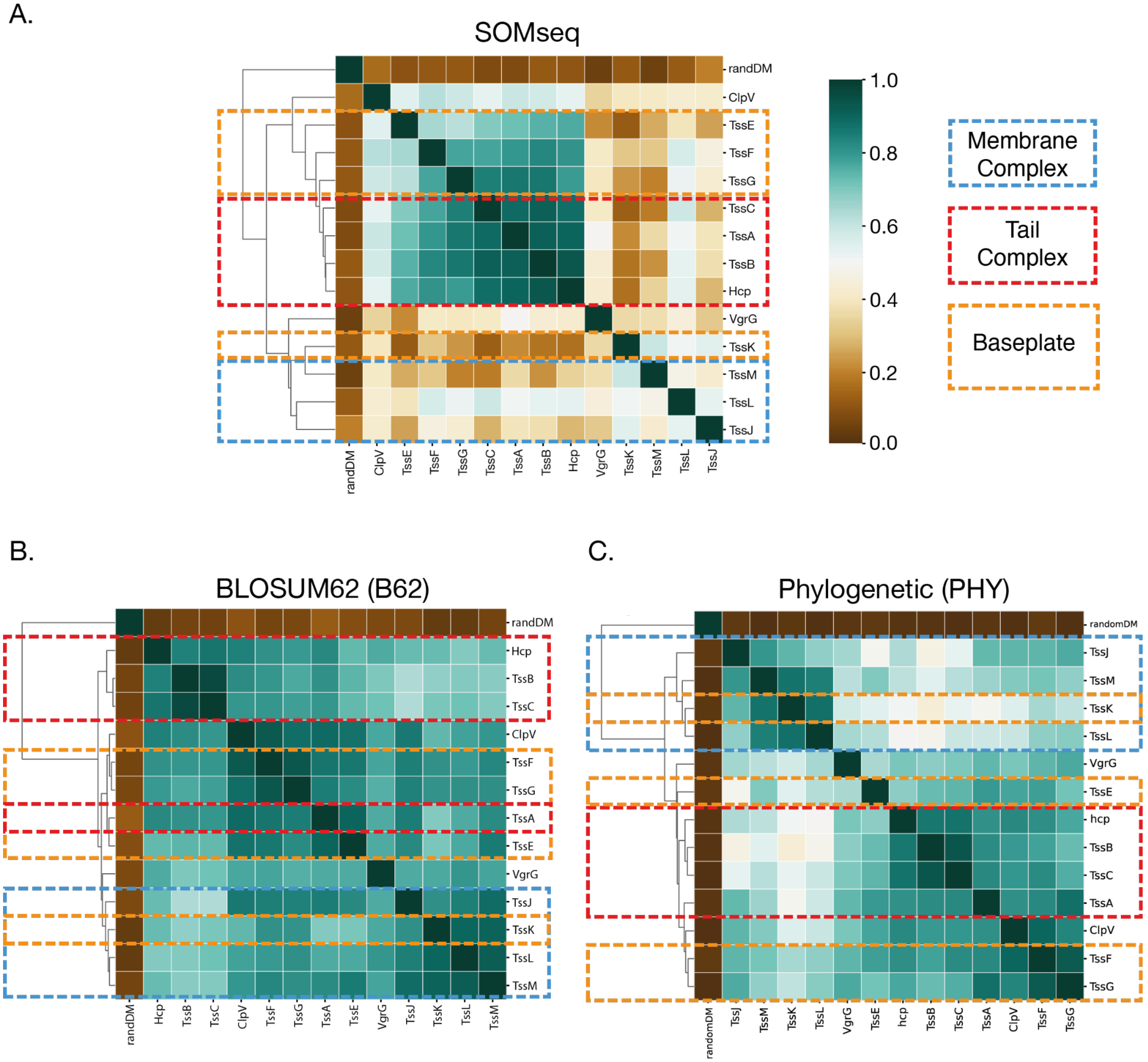

Supplementary Figure 7) T6SS correlation matrices. TC, MC, and BP clusters of correlated genes are highlighted with red, blue, and orange dashed squares, respectively. **A)** T6SS AlignScape correlation matrix. **B)** T6SS BLOSUM62 correlation matrix. **C)** T6SS phylogenetic correlation matrix.

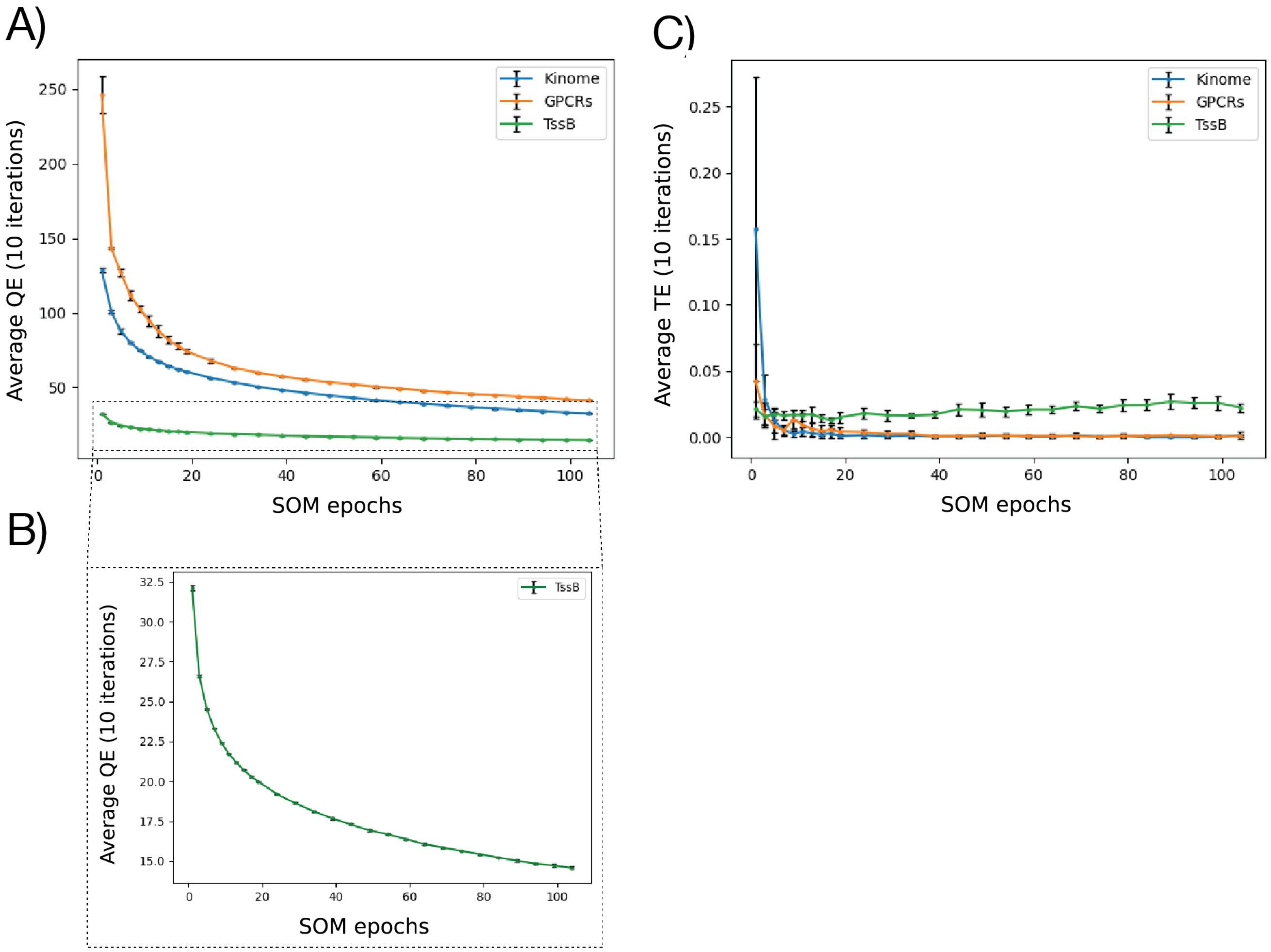

Supplementary Figure 8) Assessment of the robustness of AlignScape. A) Average SOM quantization error (QE) for a range of total epochs. B) Zoom-in into panel A TssB assessment. **B)** Average SOM topographical error (TE) for a range of total epochs. **A-C)** The total number of evaluated epochs ranged from 1 to 105, and the assessment was repeated 10 times for each epoch number. The AlignScape robustness assessment was performed with the Kinome, the GPCRs, and the TssB MSA as input.

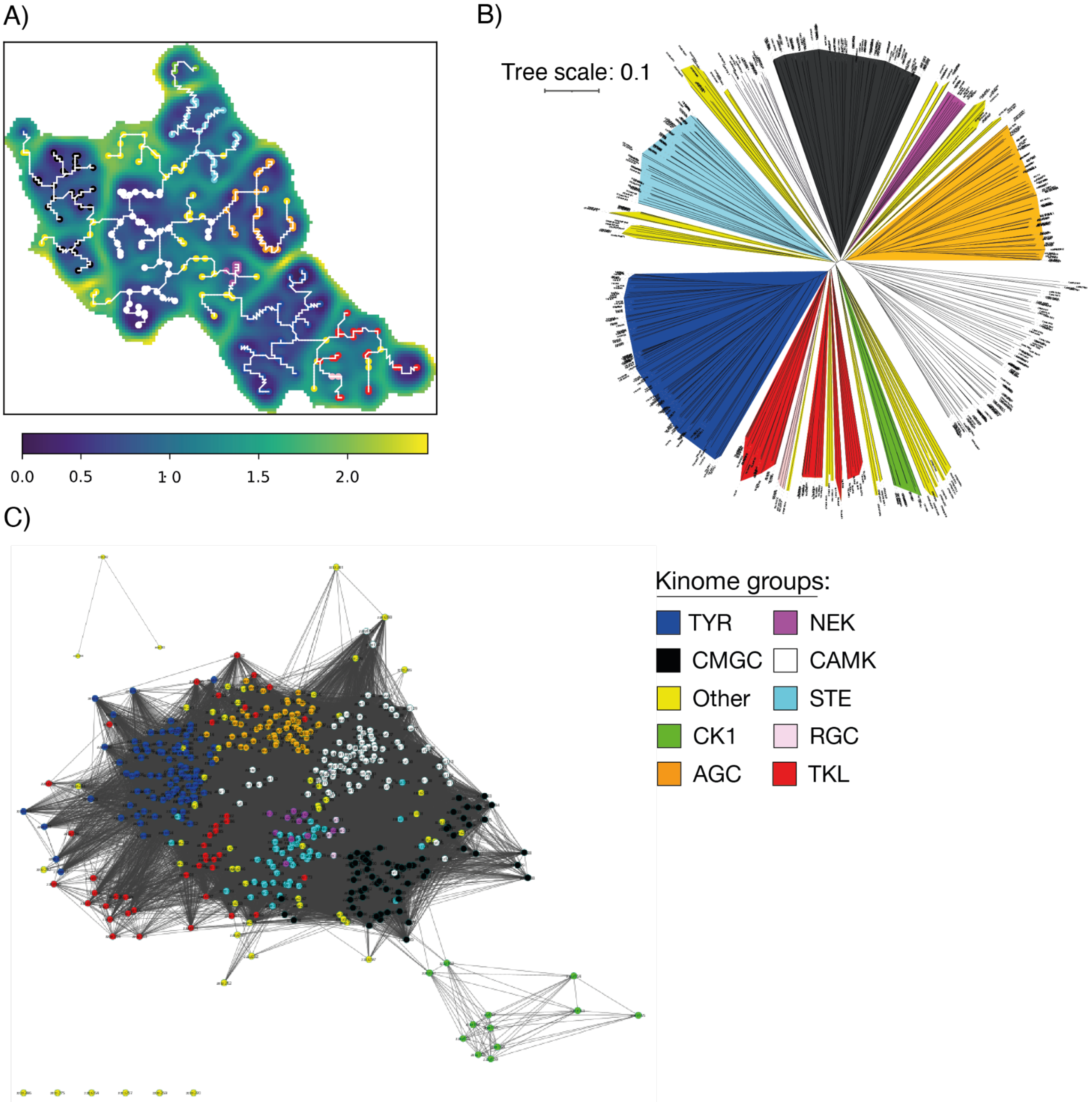

Supplementary Figure 9) Visual comparison between three representations: AlignScape U-matrix, circular unrooted phylogenetic tree, and an Sequence Similarity Network (SSN) for the human kinome sequences. A) An aperiodic U-matrix representing the sequence similarity landscape of the Human Kinome. Each sequence from the input MSA was mapped to its BMU and was color-coded based on kinase groups. **B)** Circular unrooted phylogenetic tree of human protein kinases generated using the p-distance metric between sequences, the neighbor-joining algorithm, and 1000 bootstrap replicates. These calculations were performed using the MegaX software (Kumar et al., 2018). The resulting tree was subsequently uploaded to the iTOL webserver (Letunic and Bork, 2016), where each clade was color-coded based on its corresponding kinase group. **C)** Sequence similarity network of the Human kinome. We used EFI online tools to generate the SSN (Zallot et al., 2019; Oberg et al., 2023), and the network was displayed using cytoscape (Shannon et al., 2003).

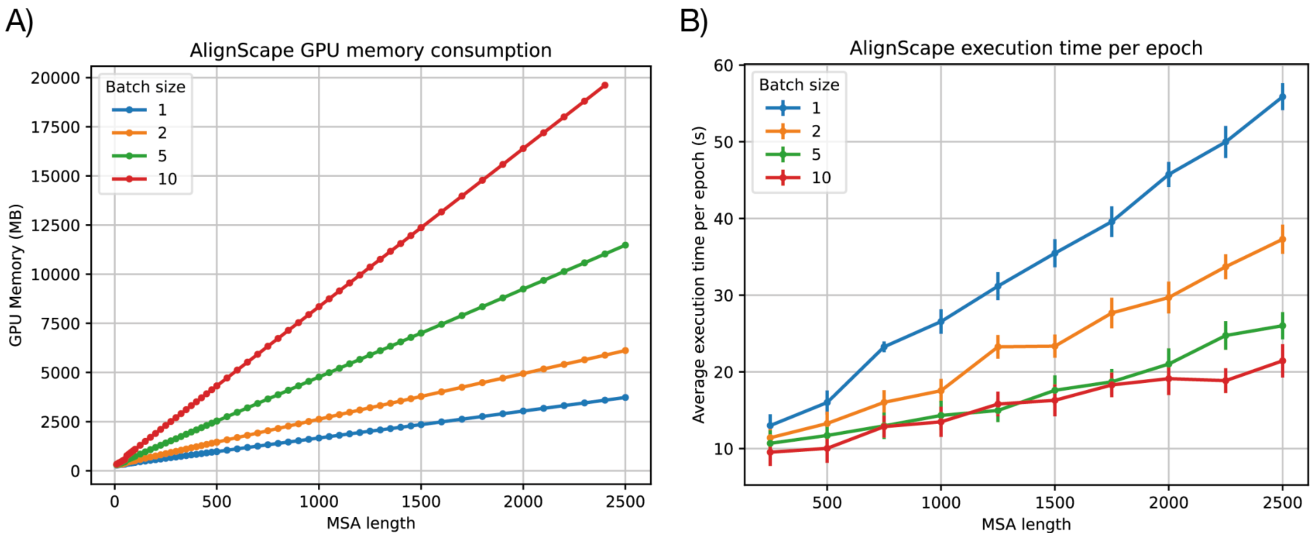

Supplementary Figure 10) AlignScape scalability. A) GPU memory allocation required for various MSA lengths and batch sizes. B) Average execution time for one training epoch across various MSA lengths and batch sizes. The average execution time and its standard deviation (depicted as error bars) were calculated based on five trials. Both GPU memory allocation and execution time tests were conducted in an NVIDIA RTX A4500 with 20GB of memory.

2.2 Supplementary Tables

Kinase	Predicted type	Kinase	Predicted type	Kinase	Predicted type	Kinase	Predicted type
AAK1	NEK	IKBKE	STE	RNASEL	STE	TLK1	CAMK
BMP2K	NEK	MLKL	TKL	RPS6KC1	AGC	TLK2	CAMK
BUB1	NEK	MOS	TKL	RPS6KL1	AGC	TP53RK	TKL
BUB1B	CAMK	NRBP1	NEK	SBK1	CAMK	TTK	CAMK
CDC7	CMGC	NRBP12	NEK	SBK2	CAMK	UHMK1	CMGC
CHUK	STE	PAN3	NEK	SBK3	CMGC	ULK1	CAMK
DSTYK	TKL	PBK	TKL	SCYL1	STE	ULK2	CAMK
EIF2AK1	NEK	PDIK1L	NEK	SCYL2	CAMK	ULK3	CAMK
EIF2AK2	NEK	PEAK1	CMGC	SCYL3	STE	ULK4	CAMK
EIF2AK3	NEK	PEAK3	TKL	STK16	NEK	WEE1	STE
EIF2AK4	STE	PIK3R4	CMGC	STK31	TKL	WEE2	STE
EIF2AK4	STE	PINK1	CAMK	STK35	NEK	WNK1	NEK
ERN1	CAMK	PKDCC	TKL	STK36	CAMK	WNK2	NEK
ERN2	CAMK	PKMYT1	STE	STKLD1	CMGC	WNK3	NEK

GAK	NEK	POMK	TKL	TBCK	TYR	WNK4	NEK
HASPIN	CMGC	PRAG1	CMGC	TBK1	STE		
IKBKB	STE	PXK	STE	TEX14	TKL		

Supplementary Table 1) Inferred kinase group for unclassified kinases (‘OTHER’ group).

A-δ seqs. in A-α branch	A-δ seqs in A-β branc	A-δ inferred seqs in A-β branc
Q8N6U8	P34981	Q9UJ42
Q96P66	Q96P65	Q8TDV5
Q9HBX8		Q8TDV2
P16473		Q9GZN0
O75473		Q99680
P22888		
Q9BXB1		
P23945		
Q9HBX9		
P47804		

Supplementary Table 2) A-δ sequences and A-δ inferred sequences outside the main A-δ branch.

GPCR	Predicted type	GPCR	Predicted type	GPCR	Predicted type
Q99678	A-delta	Q9UKP6	A-gamma	Q6W5P4	A-beta
Q8TDV2	A-delta	Q9BZJ6	A-alpha	O95800	A-beta
Q16570	A-gamma	O15552	A-delta	Q9NQS5	A-alpha
Q8NFJ6	A-beta	Q7Z601	A-delta	Q8TDS5	A-delta
Q6DWJ6	A-beta	Q99680	A-delta	Q9NS67	A-alpha
Q969V1	A-gamma	Q8TCW9	A-beta	Q99527	A-gamma
O43194	A-beta	O00270	A-delta	Q5NUL3	A-gamma
Q86SM8	A-delta	O14843	A-delta	Q8NGU9	A-beta
Q8TDV0	A-beta	Q14439	A-alpha	Q8IZ08	A-alpha
O14842	A-delta	Q96P67	A-delta	Q8TDU9	A-gamma
Q86VZ1	A-delta	Q99705	A-gamma	P60893	A-alpha
Q9NS66	A-alpha	Q8TDV5	A-delta	Q8TDU6	A-alpha
Q86SM5	A-delta	Q7Z602	A-delta	Q9UPC5	A-delta

Q9UJ42	A-alpha	Q86SP6	A-alpha	Q15760	A-beta
Q16538	A-alpha	Q96CH1	A-gamma	Q9GZN0	A-delta
Q6NV75	A-alpha	Q9Y5Y3	A-alpha	O15529	A-delta
Q8TDT2	A-gamma	Q6U736	A-alpha	Q49SQ1	A-gamma

Supplementary Table 3) Inferred GPCR A subgroups for A unclassified GPCRs (‘A-other’ label).

Organism	Gene Cluster ID	Kegg Org.	NCBI Tax.	T6SS subtype	Start Pos.	End Pos.
Enteroaggregative Escherichia Coli	EAEC1	eck	585055	i1	241895	263191
	EAEC2	eck	585055	i4b	3369231	3387598
	EAEC3	eck	585055	i2	3394967	3428639
Pseudomonas Aeruginosa	P. a1	paei	1367493	i3	90844	117524
	P. a2	paei	1367493	i1	1803621	1822596
	P. a3	paei	1367493	i4b	2607127	2626205
Acinetobacter Baumannii	A. b	abaz	1400867	i4b	2353802	2383994
Vibrio Cholerae	V. c	vcm	579112	i1	115133	141582
Serratia Marcescens	S.m1	serf	1327989	i3	2826850	2855641
	S.m2	serf	1327989	i3	3025547	3063156
Salmonella Enterica Serovar Typhimurium	S.tm	setc	1271862	i3	3228066	3262201
Klebsiella Pneumonia	K.p1	kpu	484021	i2	2268445	2329098
	K.p2	kpu	484021	i2	3185286	3215355
Aeromonas Hydrophila	A.h	ahj	1419584	i1	2871129	2903427
Campylobacter Jejuni	C.j	cjl	32022	i1	964886	992379
Yersinia Enterocolitica	Y.e	yet	630	i3	3532481	3566567
Yersinia Pseudotuberculosis	Y.ps1	ypi	349747	i1	361733	385634
	Y.ps2	ypi	349747	i2	884659	901538
	Y.ps3	ypi	349747	i2	932854	975730

	Y.ps4	ypi	349747	i3	1587097	1619289
	Y.ps5	ypi	349747	i2	2807319	2847574
	Y.ps6	ypi	349747	i3	3850258	3875321
Yersinia Pestis	Y.p1	ype	214092	i3	531628	556690
	Y.p2	ype	214092	i2	1072398	1089082
	Y.p3	ype	214092	i2	1653684	1689928
	Y.p4	ype	214092	i3	3050040	3065993
	Y.p5	ype	214092	i3	3277633	3302045
	Y.p6	ype	214092	i1	4001721	4035636
Edwardsiella Tarda	E.t	etr	498217	i4b	2554393	2580320
Agrobacterium Tumefaciens	A.t	atf	358	i5	1998442	2033793
Burkholderia Cenocepacia	B.c1	bceo	1055524	i2	172983	205070
	B.c2	bceo	1055524	i4b	347141	370099
Burkholderia Mallei	B.m1	bmal	13373	i3	161777	187715
	B.m2	bmal	13373	i3	793604	820431
	B.m3	bmal	13373	i4b	942176	959096
	B.m4	bmal	13373	i3	972744	1000481
	B.m5	bmal	13373	i1	1794749	1822794
Burkholderia Pseudomallei	B.ps1	bps	272560	i3	115016	146918
	B.ps2	bps	272560	i3	218043	251322
	B.ps3	bps	272560	i3	695254	729898
	B.ps4	bps	272560	i1	2032502	2067066
	B.ps5	bps	272560	i3	2829708	2858941
	B.ps6	bps	272560	i4b	3694948	3719234
Xanthomonas Citri	X.c	xac	190486	i3	4818638	4875351

Pseudomonas fluorescens	P.f	pfn	294	i1	1170803	1193050
Paraburkholderia phymatum	P.p1	bph	391038	i4a	510771	535444
	P.p2	bph	391038	i3	661145	674037
	P.p3	bph	391038	i3	1673089	1686527
Rhizobium leguminosarum	R.l	rlb	754523	i5	176094	196011
Vibrio parahaemolyticus	V.p1	vpa	223926	i5	1081544	1101710
	V.p2	vpa	223926	i1	1495971	1522017
Ralstonia solanacearum	R.s	rso	267608	i4b	929249	965066
Xanthomonas Phaseoli	X.p	xph	317013	i3	3060118	3120563
Citrobacter freundii	C.f1	cfd	1333848	i2	446079	452361
	C.f2	cfd	1333848	i4a	1826997	1839918
Helicobacter hepaticus	H.h	hhe	235279	i1	234543	246791

Supplementary Table 4) List of T6SS gene clusters. It includes information about the organism of origin, the gene cluster acronym, the organism TaxID in both KEGG and NCBI database, and the starting and ending nucleotide.

T6SS Protein/Domain	Maximum Likelihood model
TssA	LG+F+G4
TssB	LG+G4
TssC	LG+F+I+G4
TssE	LG+F+I+G4
TssF	LG+F+I+G4
TssG	LG+F+I+G4
TssJ	LG+F+I+G4
TssK	LG+F+G4
TssL	LG+F+I+G4
TssM	LG+F+I+G4
hcp	LG+G4
VgrG	LG+F+I+G4

ClpV	LG+F+I+G4

Supplementary Table 5) List of T6SS core components. It includes the T6SS gene and the maximum likelihood model utilized to generate the gene phylogenetic tree.

Supplementary Table 6) Inferred T6SS[i] subtype for TssB unclassified sequences ('unk' label).

3 References

Akaike, H. (1998). "A New Look at the Statistical Model Identification," in *Selected Papers of Hirotugu Akaike* Springer Series in Statistics., eds. E. Parzen, K. Tanabe, and G. Kitagawa (New York, NY: Springer), 215–222. doi: 10.1007/978-1-4612-1694-0_16.

Bouvier, G., Desdouits, N., Ferber, M., Blondel, A., and Nilges, M. (2015). An automatic tool to analyze and cluster macromolecular conformations based on self-organizing maps. *Bioinforma. Oxf. Engl.* 31, 1490–1492. doi: 10.1093/bioinformatics/btu849.

Cherrak, Y., Filella-Merce, I., Schmidt, V., Byrne, D., Sgoluppi, V., Chaiaheloudjou, R., et al. (2021). Inhibiting Type VI Secretion System Activity with a Biomimetic Peptide Designed To Target the Baseplate Wedge Complex. *mBio* 12, e0134821. doi: 10.1128/mBio.01348-21.

Chiner-Oms, A., and González-Candelas, F. (2016). EvalMSA: A Program to Evaluate Multiple Sequence Alignments and Detect Outliers. *Evol. Bioinforma. Online* 12, 277–284. doi: 10.4137/EBO.S40583.

ClipKIT: A multiple sequence alignment trimming software for accurate phylogenomic inference | PLOS Biology (n.d.). Available at: https://journals.plos.org/plosbiology/article?id=10.1371/journal.pbio.3001007 [Accessed December 5, 2022].

Cvicek, V., Goddard, W. A., and Abrol, R. (2016). Structure-Based Sequence Alignment of the Transmembrane Domains of All Human GPCRs: Phylogenetic, Structural and Functional Implications. *PLoS Comput. Biol.* 12, e1004805. doi: 10.1371/journal.pcbi.1004805.

Gouy, M., Guindon, S., and Gascuel, O. (2010). SeaView version 4: A multiplatform graphical user interface for sequence alignment and phylogenetic tree building. *Mol. Biol. Evol.* 27, 221–224. doi: 10.1093/molbev/msp259.

Kalyaanamoorthy, S., Minh, B. Q., Wong, T. K. F., von Haeseler, A., and Jermiin, L. S. (2017). ModelFinder: fast model selection for accurate phylogenetic estimates. *Nat. Methods* 14, 587–589. doi: 10.1038/nmeth.4285.

Kanehisa, M., and Goto, S. (2000). KEGG: kyoto encyclopedia of genes and genomes. *Nucleic Acids Res.* 28, 27–30. doi: 10.1093/nar/28.1.27.

Katoh, K., Misawa, K., Kuma, K., and Miyata, T. (2002). MAFFT: a novel method for rapid multiple sequence alignment based on fast Fourier transform. *Nucleic Acids Res.* 30, 3059–3066. doi: 10.1093/nar/gkf436.

Kumar, S., Stecher, G., Li, M., Knyaz, C., and Tamura, K. (2018). MEGA X: Molecular Evolutionary Genetics Analysis across Computing Platforms. *Mol. Biol. Evol.* 35, 1547–1549. doi: 10.1093/molbev/msy096.

Letunic, I., and Bork, P. (2016). Interactive tree of life (iTOL) v3: an online tool for the display and annotation of phylogenetic and other trees. *Nucleic Acids Res.* 44, W242-245. doi: 10.1093/nar/gkw290.

Li, J., Yao, Y., Xu, H. H., Hao, L., Deng, Z., Rajakumar, K., et al. (2015). SecReT6: a web-based resource for type VI secretion systems found in bacteria. *Environ. Microbiol.* 17, 2196–2202. doi: 10.1111/1462-2920.12794.

Li, W., Jaroszewski, L., and Godzik, A. (2001). Clustering of highly homologous sequences to reduce the size of large protein databases. *Bioinformatics* 17, 282–283. doi: 10.1093/bioinformatics/17.3.282.

Madden, T., and Camacho, C. (2021). *BLAST+ features*. National Center for Biotechnology Information (US) Available at: https://www.ncbi.nlm.nih.gov/books/NBK569839/ [Accessed December 5, 2022].

Mallet, V., Nilges, M., and Bouvier, G. (2021). quicksom: Self-Organizing Maps on GPUs for clustering of molecular dynamics trajectories. *Bioinforma. Oxf. Engl.* 37, 2064–2065. doi: 10.1093/bioinformatics/btaa925.

Modi, V., and Dunbrack, R. L. (2019). A Structurally-Validated Multiple Sequence Alignment of 497 Human Protein Kinase Domains. *Sci. Rep.* 9, 19790. doi: 10.1038/s41598-019-56499-4.

Mount, N. J., and Weaver, D. (2011). Self-organizing maps and boundary effects: quantifying the benefits of torus wrapping for mapping SOM trajectories. *Pattern Anal. Appl.* 14, 139–148. doi: 10.1007/s10044-011-0210-5.

Oberg, N., Zallot, R., and Gerlt, J. A. (2023). EFI-EST, EFI-GNT, and EFI-CGFP: Enzyme Function Initiative (EFI) Web Resource for Genomic Enzymology Tools. *J. Mol. Biol.* 435, 168018. doi: 10.1016/j.jmb.2023.168018.

Pedregosa, F., Varoquaux, G., Gramfort, A., Michel, V., Thirion, B., Grisel, O., et al. (2011). Scikit-learn: Machine Learning in Python. *J. Mach. Learn. Res.* 12, 2825–2830.

Shannon, P., Markiel, A., Ozier, O., Baliga, N. S., Wang, J. T., Ramage, D., et al. (2003). Cytoscape: a software environment for integrated models of biomolecular interaction networks. *Genome Res.* 13, 2498–2504. doi: 10.1101/gr.1239303.

Sonnhammer, E. L., and Hollich, V. (2005). Scoredist: A simple and robust protein sequence distance estimator. *BMC Bioinformatics* 6, 108. doi: 10.1186/1471-2105-6-108.

Suzek, B. E., Wang, Y., Huang, H., McGarvey, P. B., Wu, C. H., and the UniProt Consortium (2015). UniRef clusters: a comprehensive and scalable alternative for improving sequence similarity searches. *Bioinformatics* 31, 926–932. doi: 10.1093/bioinformatics/btu739.

Talevich, E., Invergo, B. M., Cock, P. J., and Chapman, B. A. (2012). Bio.Phylo: A unified toolkit for processing, analyzing and visualizing phylogenetic trees in Biopython. *BMC Bioinformatics* 13, 209. doi: 10.1186/1471-2105-13-209.

Virtanen, P., Gommers, R., Oliphant, T. E., Haberland, M., Reddy, T., Cournapeau, D., et al. (2020). SciPy 1.0: fundamental algorithms for scientific computing in Python. *Nat. Methods* 17, 261–272. doi: 10.1038/s41592-019-0686-2.

Walt, S. van der, Schönberger, J. L., Nunez-Iglesias, J., Boulogne, F., Warner, J. D., Yager, N., et al. (2014). scikit-image: image processing in Python. *PeerJ* 2, e453. doi: 10.7717/peerj.453.

Waskom, M., Botvinnik, O., O'Kane, D., Hobson, P., Lukauskas, S., Gemperline, D. C., et al. (2017). mwaskom/seaborn: v0.8.1 (September 2017). doi: 10.5281/zenodo.883859.

Zallot, R., Oberg, N., and Gerlt, J. A. (2019). The EFI Web Resource for Genomic Enzymology Tools: Leveraging Protein, Genome, and Metagenome Databases to Discover Novel Enzymes and Metabolic Pathways. *Biochemistry* 58, 4169–4182. doi: 10.1021/acs.biochem.9b00735.
